# Quantum‐Mechanically Refined, Dynamics‐Coupled, and AI‐Augmented Elucidation of Epigenetic Inhibition: An In Silico Paradigm Targeting HDAC8 of *Schistosoma mansoni*

**DOI:** 10.1155/jotm/1172449

**Published:** 2025-12-22

**Authors:** Mohd Imran, Talha Jawaid, Hayaa M. Alhuthali, Amani A. Alrehaili, Abdullah R. Alzahrani, Zia Ur Rehman, Elliot Mbunge, Tafadzwa Dzinamarira

**Affiliations:** ^1^ Center for Health Research, Northern Border University, Arar, 73213, Saudi Arabia, nbu.edu.sa; ^2^ Department of Pharmacology, College of Medicine, Imam Mohammad Ibn Saud Islamic University (IMSIU), Riyadh, Saudi Arabia, imamu.edu.sa; ^3^ Department of Clinical Laboratory Sciences, College of Applied Medical Sciences, Taif University, P.O. Box 11099, Taif, 21944, Saudi Arabia, tu.edu.sa; ^4^ Department of Pharmacology and Toxicology, Faculty of Medicine, Umm Al-Qura University, P.O. Box 13578 Al-Abidiyah, Makkah, 21955, Saudi Arabia, uqu.edu.sa; ^5^ Health Research Centre, Jazan University, P.O. Box 114, Jazan, 45142, Saudi Arabia, jazanu.edu.sa; ^6^ Department of Pharmaceutical Chemistry and Pharmacognosy, Faculty of Pharmacy, Jazan University, P.O. Box 114, Jazan, 45142, Saudi Arabia, jazanu.edu.sa; ^7^ Department of Applied Information Systems, University of Johannesburg, Johannesburg, South Africa, uj.ac.za; ^8^ School of Health Systems and Public Health, University of Pretoria, Pretoria, South Africa, up.ac.za; ^9^ ICAP at Columbia University, Lusaka, Zambia

**Keywords:** DFT, HDAC8, machine learning, *Schistosoma mansoni*, schistosomiasis

## Abstract

The increasing burden of schistosomiasis, compounded by the restriction imposed by monotherapeutic regimens, highlights the pressing need for new molecules that target specific molecular pathways. *Schistosoma mansoni* histone deacetylase 8 (SmHDAC8), a zinc‐dependent epigenetic regulator, has emerged as a nonredundant and druggable enzyme, critical for parasite survival, fertility, and chromatin homeostasis. In this study, we outline multiple‐mode computational analysis involving structure‐based virtual screening against a chemically diverse ligand library, frontier molecular orbital analysis through DFT, large‐scale molecular dynamics (MD) simulations (500 ns), and molecular mechanics/gas‐phase/Generalized Born (MM/GBSA) energy component analysis, complemented with machine learning–guided pIC_50_ model building and prediction. Our screening cascade comprising docking, MM, and MD identified the lead candidate, **24374890**, with the best docking score (−9.5 kcal/mol) and desirable electronic configuration (HOMO–LUMO gap: 4.143 eV) for its optimal reactivity–stability balance. MD simulations confirmed its stability in the short term, as well as its conformational preservation in the SmHDAC8 catalytic pocket, as evidenced through low RMSD values, stable free energy basins, and sustained intermolecular interactions. Hydrogen bond analysis proved that compounds 24374890 and 24280440 kept 1–4 stable hydrogen bonds for the entire 500 ns simulation, supporting their strong and stable binding in SmHDAC8’s active site. Thermodynamic calculations through MM/GBSA indicated **24374890** has the best energetics for binding (ΔG_total = −65.11 kcal/mol), comprising largely van der Waals and nonpolar solvation energies. Finally, the pIC_50_ value for **24374890** was predicted, through supervised machine learning, as 8.1, better than the reference molecule. These convergent findings from quantum mechanical, molecular mechanical, and AI‐based computations validate **24374890** as an SmHDAC8 inhibitor that is structurally and dynamically sound. These calculations need to be supported with in vitro enzyme inhibition experiments against recombinant SmHDAC8 and cytotoxicity profiling in schistosome cultures. Moreover, X‐ray crystallography or cryo‐EM analysis of the SmHDAC8–**24374890** complex would reveal detailed binding conformations.

## 1. Introduction

Schistosomiasis is still one of the most debilitating of the neglected tropical diseases, infecting more than 240 million individuals globally and putting an estimated 800 million at risk, mostly in sub‐Saharan Africa, certain regions of South America, the Middle East, and parts of Southeast Asia [1]. *Schistosoma mansoni* is among the significant causative agents of intestinal schistosomiasis and continues as a significant public health issue [2]. Global analyses describe its epidemiology, pathology, and the limitations of current control strategies, highlighting the urgent need for improved interventions [2]. Although praziquantel remains the mainstay of treatment, detailed studies on its metabolism, mode of action, and clinical performance have revealed reduced efficacy in juvenile worms and early evidence of drug resistance in certain regions [3, 4].

Recent WHO guidelines recommend a more integrated approach that combines preventive chemotherapy with snail control, improved water, sanitation, and hygiene (WASH), and enhanced surveillance to strengthen long‐term impact [5]. Mass drug administration (MDA) remains a cornerstone of these programs, successfully reducing prevalence in many endemic areas, yet alone it is insufficient to achieve elimination without complementary public health measures [6]. Evidence from multiple endemic settings underscores the urgency of addressing the limitations of praziquantel through diversification of therapeutic options [4]. National programs, such as the comprehensive elimination campaign in Rwanda, demonstrate the effectiveness of coordinated mapping, targeted treatment, and sustained monitoring in progressing toward elimination goals [7].

In parallel, adopting a One Health framework that considers human, animal, and environmental health has been emphasized as essential for sustainable schistosomiasis control. This approach addresses zoonotic transmission cycles and environmental reservoirs alongside human treatment, providing a broader platform for achieving WHO elimination targets [8]. Children carry the highest burden in endemic areas, suffering from long‐term sequelae such as anemia, growth delay, and cognitive impairments [9]. The disease also presents economic issues, decreasing working capacity and putting an overload on health care resources.

Epigenetic regulation in schistosomes has been identified as a key driver of parasite development, reproduction, and survival, positioning it as a promising therapeutic target [10]. Disruption of these mechanisms can impair egg formation and reduce transmission potential [11]. In the snail intermediate host, epigenetic plasticity influences susceptibility to infection, highlighting new avenues for control [12]. Reviews of current knowledge reveal substantial progress but also critical gaps requiring further investigation [13]. Specific epigenetic markers have been linked to schistosomiasis, offering potential tools for diagnosis and targeted therapy [14]. Chromatin regulation and histone‐modifying enzymes, in particular, show strong potential as novel drug targets [15].

Among the different isoforms of HDAC, one of them, namely, *Schistosoma mansoni* histone deacetylase 8 (SmHDAC8), has been of particular interest owing to its structural specificity, substrate choice, and drugability [16–18]. SmHDAC8, unlike its counterparts, contains specific amino acid residues in the catalytic pocket, which may be an entry point to selectively target it without cross‐reacting with host HDACs [16, 17, 19]. The biological significance of SmHDAC8 is also reinforced by studies at the genetic and biochemical levels [20]. Silencing or pharmacological inhibition of SmHDAC8 perturbs parasite development, alters gene expression associated with survival and reproduction, and causes phenotypic defects, thus establishing it as a target for drug development [21, 22]. SmHDAC8 is significant in stem cell viability and tegument integrity, two processes that are paramount to the survivability of the parasite in the bloodstream of the host [23]. Lack of upregulation of other HDACs in SmHDAC8‐suppressed lines further attests to its nonredundant role [15, 24]. Additionally, advancing structural information, such as crystal structures of SmHDAC8 in bound form to inhibitors, has facilitated structure‐guided drug development, presenting an accessible paradigm for designing selective therapeutics [16, 25].

In spite of advances in this area, treatment of schistosomiasis is still bound by therapeutic restraints. Praziquantel, which remains the only recommended treatment by WHO, is limited in potency against immature larval stages and fails to safeguard against reinfection [3, 26]. Resistance is not yet prevalent but is increasingly of concern, especially where there is extensive use of MDA programs [6, 27]. Various small molecules and epigenetic inhibitors have also been investigated for antischistosomal activities. For instance, compounds like J1075 and PCI‐34051, which were initially designed for HDAC8 in humans, proved to be potent antischistosomal agents, but their selectivity and in vivo safety remain debatable [16, 25, 28, 29]. Other scaffolds of hydroxamic acids and benzamides have also been repurposed or structurally optimized to target SmHDAC8, though most of these prospects remain at or below in vitro advances [18, 30]. Hence, identification of potent, safe, and selective SmHDAC8 inhibitors remains an area of high‐priority interest in drug development against schistosomiasis.

Due to the constraints imposed by existing schistosomiasis therapy as well as growing praziquantel resistance, a severe necessity exists for new therapeutic targets as well as drug leads. SmHDAC8 represents a nonredundant, structurally distinct enzyme vital for parasite survival that can be positioned as a promising target for selective inhibitor molecules. The hypothesis here is that the structural distinctiveness of SmHDAC8 compared to human HDACs can be exploited for selective drug development. To investigate this further, we undertook a multifaceted computer‐aided approach involving structure‐centered virtual screening, quantum mechanic computations, and molecular dynamic simulation, as well as machine‐learned guided prediction of bioactivity for finding and validating new selective SmHDAC8 inhibitors.

**Figure 1:**
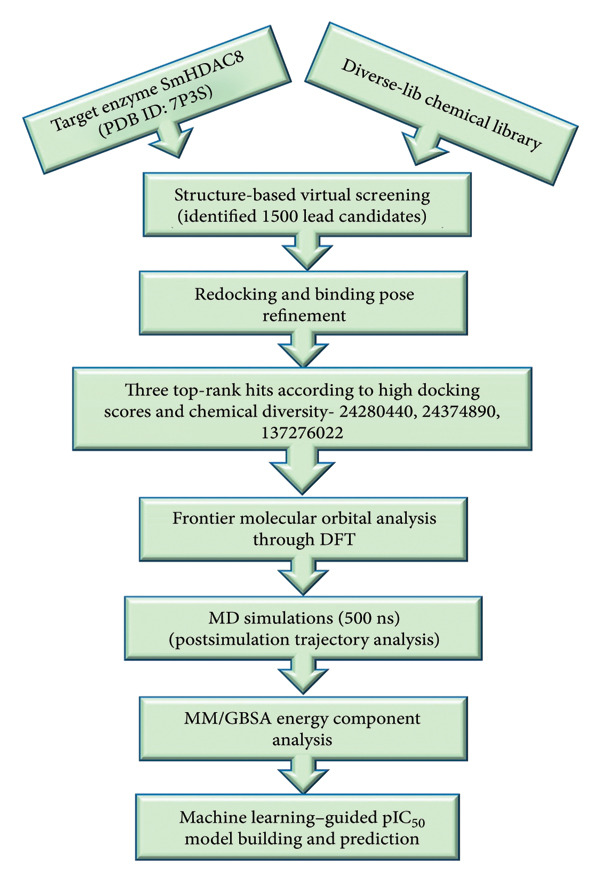
Workflow summarizing the computational methodology used in this study.

Considering increasing interest in epigenetic targets and an immediate need for novel therapeutics, our research aimed to find effective inhibitors of *Schistosoma mansoni* HDAC8 using a structure‐based, rational drug design strategy. The aim was to utilize both data‐driven predictive modeling and principles of quantum chemistry to identify novel chemotypes of high affinity and biologic relevance against SmHDAC8. With an eye on the structural novelty of SmHDAC8 and the use of chemically diverse scaffolds, this research hopes to provide novels amenable to further development and optimization. A detailed workflow illustrating the overall computational approach is presented in Figure 1.

## 2. Results

The virtual screening against SmHDAC8 identified 1500 lead candidates from the Diverse‐Lib chemical library with docking scores from −9.5 to −7.0 kcal/mol, indicative of favorable protein–ligand interactions in the target enzyme catalytic domain as shown in the supporting file as Table S1. The workflow for docking subjected the ligands to stringent filtering parameters, ensuring only those ligands with desirable physicochemical properties and good docking scores were filtered for advanced analysis. Of the screened dataset, three compounds—**24374890**, **137276022**, and **24280440**—were top‐rank hits according to their high docking scores and chemical diversity. The ligands performed better than most in the dataset and were shortlisted for further analysis. As reference controls for comparing the predictive accuracy in the docking assays, known HDAC8‐targeting compounds were included in parallel.

This result demonstrates the chemical diversity and bindability of the Diverse‐Lib database and lends credence to the belief that previously undiscovered scaffolds might be able to yield selective affinity toward the parasitic HDAC8 targets. The top three candidates were chosen based not only on energetic aspects but also on structural differentiation, paving the way for quantum mechanical calculations and dynamic simulations in the following stages of the work.

### 2.1. Density Functional Theory (DFT)

Frontier molecular orbital (FMO) analysis of the chosen ligands was achieved through the application of the DFT in order to determine their electronic distribution and tendencies toward reactivity. Highest occupied molecular orbital (HOMO) and lowest unoccupied molecular orbital (LUMO) isosurfaces of **24374890**, **137276022**, **24280440**, and the reference molecule appear in Figures 2(a), 2(b), 2(c), 2(d), 2(e), 2(f), 2(g), 2(h), whereas their orbital energies and gaps in energy appear in the provided dataset.

Figure 2Frontier molecular orbitals of top screened ligands. Panels (a–h) display HOMO and LUMO surfaces for (a–b) 24374890, (c–d) 137276022, (e–f) 24280440, and (g–h) the reference compound.

**Figure:**
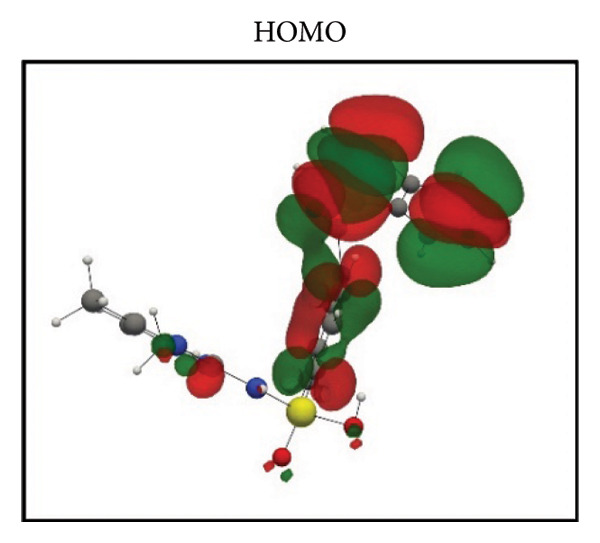
(a)

**Figure:**
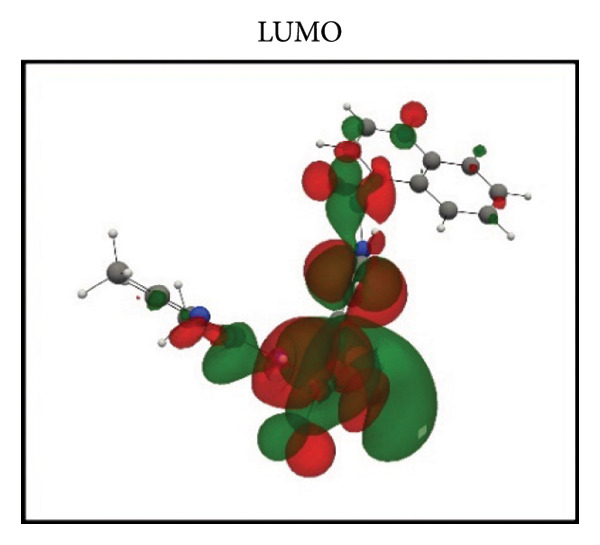
(b)

**Figure:**
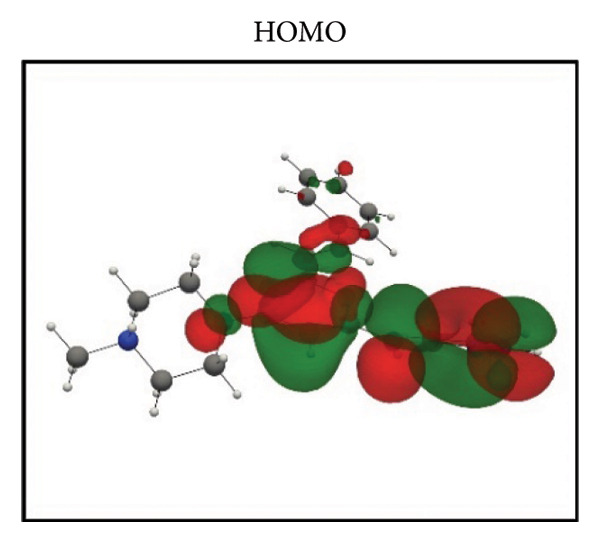
(c)

**Figure:**
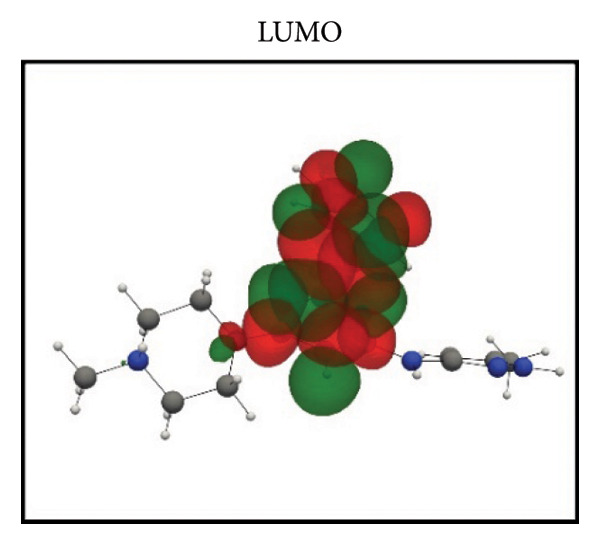
(d)

**Figure:**
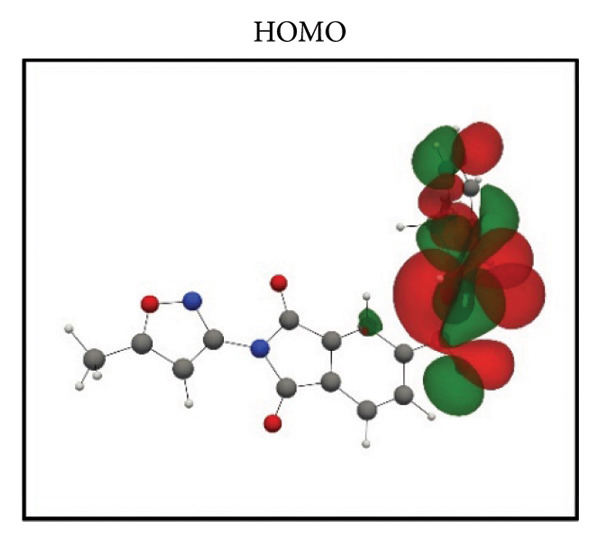
(e)

**Figure:**
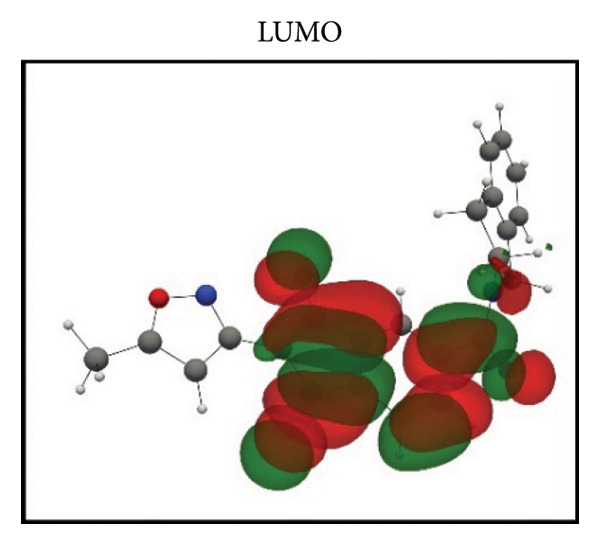
(f)

**Figure:**
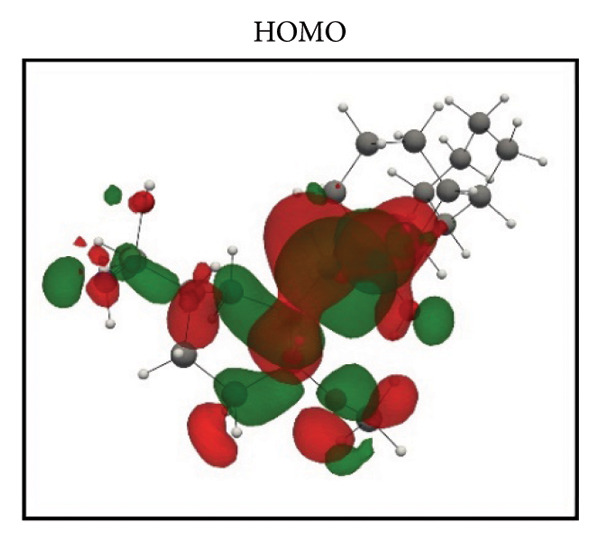
(g)

**Figure:**
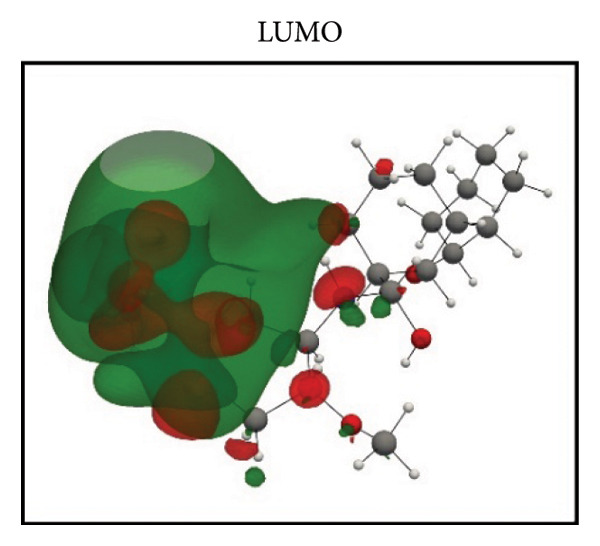
(h)

**24374890** exhibited HOMO energy and LUMO energy values as −0.217776 Hartree and −0.065524 Hartree, respectively, with the HOMO–LUMO gap being 4.143 eV (Figures 2(a), 2(b)). HOMO density was extensively delocalized throughout the sulfur‐containing moiety and the extended *π*‐system, implying ready electrophile attack points, and LUMO was localized over the terminal aromatics, implying probable nucleophile attack positions.

**137276022** possessed an intrinsically narrower energy gap of 3.790 eV, having HOMO and LUMO energies at the level of −0.186038 and −0.046775 Hartree, respectively (Figures 2(c), 2(d)). The relatively narrower gap indicates higher chemical softness and, probably, greater biologic reactivity. Charge delocalization patterns reveal high conjugated segment overlap, signifying high electron transport properties.

**24280440**, with the lowest gap value of 3.503 eV among the test compounds, indicates greater potential for charge transfer interactions (Figures 2(e), 2(f)). The HOMO energy was at −0.237295 Hartree, while the LUMO was at −0.108578 Hartree. Spatial distribution shows an extensive *π*‐cloud through the center framework, facilitating its interaction with electrophilic residues in the pocket.

Whereas, the reference molecule showed an appreciably large HOMO–LUMO gap of 6.396 eV, having a positive LUMO energy (+0.030079 Hartree), reflecting less reactivity and higher electronic stability (Figures 2(g), 2(h). The visible gap energy difference between the reference and the test compounds reflects the higher chemical flexibility of the filtered candidates.

### 2.2. Molecular Redocking and Interaction Analysis

**Figure 3:**
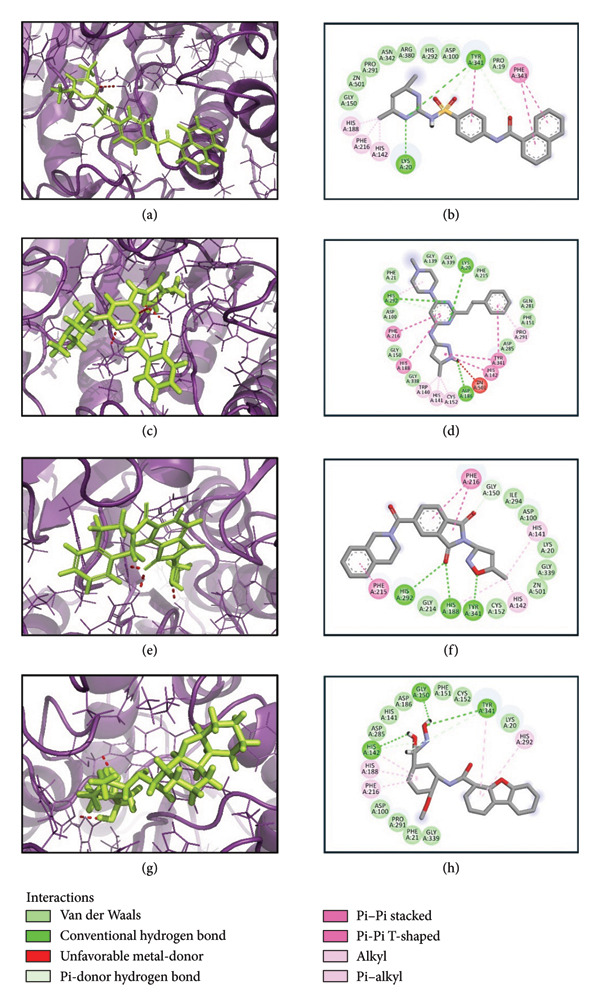
3D and 2D interaction analysis of selected compounds and control within the active site of the target protein. Panels (a–h) represent the binding interactions of compounds (a–b) 24374890, (c–d) 137276022, (e–f) 24280440, and (g–h) the control.

The selected ligands underwent redocking at exhaustiveness = 8 after DFT optimization to enhance their binding pose and ensure spatial compatibility at the active site. The renewed docking scores confirmed the high affinity of the ligands, with **24374890**, **137276022**, and **24280440** having docking scores of −9.5, −9.0, and −8.5 kcal/mol, respectively, better than the reference compound, whose docking score was −7.4 kcal/mol. Also, to test sampling robustness, all docking runs were repeated with exhaustiveness = 100. The rank order, binding poses, and docking scores were identical to those obtained at exhaustiveness = 8 (24374890: −9.5; 137276022: −9.0; 24280440: −8.5; reference: −7.4 kcal/mol), confirming the robustness of the docking outcomes. Postoptimal docking indicates an increase in geometric fidelity and electronic consistency of the ligands with the target pocket shown in Figures 3(a), 3(b), 3(c), 3(d), where 3D structures were generated via PyMOL and 2D through BIOVIA, and their interactions are given in Table 1 [31, 32]. To validate the docking protocol, the co‐crystallized inhibitor from PDB ID: 7P3S was redocked into the SmHDAC8 active site, and the predicted pose was superimposed with the experimental structure using UCSF Chimera software. The resulting RMSD of 0.7 Å confirmed accurate pose reproduction and validated the docking methodology (Supporting Figure S4).

**Table 1:** Illustrating the key binding interactions for selected ligand–protein complexes.

S. no.	Complex	H‐bond	Hydrophobic	π–π stacking/π–π cation
1	24374890	Lys^20^, Tyr^341^	Pro^19^, Asp^100^, Gly^150^, Pro^291^, His^292^, Asn^342^, Arg^380^, ZN^501^	His^142^, His^188^, Phe^216^, Tyr^341^, (Phe^343^)^2^
2	137276022	Lys^20^, Asp^186^, His^292^	Phe^21^, Asp^100^, Gly^139^, Gly^150^, Phe^151^, Phe^215^, Gln^281^, Asp^285^, Gly^338^, Gly^339^	Trp^140^, His^141^, His^142^, Pro^291^, Cys^152^, His^188^, Phe^216^, His^292^, (Tyr^341^)^2^
3	24280440	His^188^, His^292^, Tyr^341^,	Lys^20^, Asp^100^, Cys^152^, Gly^214^, Ile^294^, Gly^339^, ZN^501^	His^142^, His^141^, His^188^, Phe^215^, (Phe^216^)^2^
5	Control	His^14^, Gly^150^,Tyr^341^	Lys^20^, Phe^21^, Asp^100^, His^141^, Phe^151^, Cys^152^, Asp^186^, Asp^285^, Pro^291^, Gly^339^	His^142^, His^188^, Phe^216^, His^292^, Tyr^341^

*Note:* Interactions are listed for compounds (a) 24374890, (b) 137276022, (c) 24280440, and (d) the control.

Intermolecular interaction analysis indicated all investigated complexes had multiple stabilization interactions within the protein’s active pocket, yet of different intensities and residue involvement. Complex 24374890 showed strong stabilization by hydrogen bonds to Lys20 and Tyr341, assisted by a tight network of hydrophobic residues (Pro19, Asp100, Gly150, Pro291, His292, Asn342, and Arg380) and π–π stacking by His142, His188, Phe216, Tyr341, and Phe343, indicating rigid anchoring around the zinc catalytic Zn^2+^ ion (Figure 3(a)). Complex 137276022 exhibited several hydrogen bonds by Lys20, Asp186, and His292, accompanied by widespread hydrophobic contacts (Phe21, Asp100, Gly139, Phe151, Phe215, Gln281, Asp285, Gly338, and Gly339) and aromatic π–π stacking by Trp140, His141, Phe216, and Tyr341, indicating improved ligand accommodation and strong aromatic stabilization, hence an indication of large stabilization enthalpy and high affinity for proteins by the mechanism of increased accessible surface contact and aromatic stabilization assistance, respectively (Figure 3(b)). Complex 24280440 also possessed a strong interaction profile, having hydrogen bonds by His188, His292, and Tyr341 and establishing hydrophobic and π–π contacts by key residues (Lys20, Asp100, Cys152, Gly214, Ile294, Gly339, His142, His141, and Phe216) around ZN501 (Figure 3(c)). The control complex, despite retaining crucial interactions like hydrogen bonds by His14, Gly150, and Tyr341, exhibited comparatively weak hydrophobic coverage, hence less stability (Figure 3(d)). Generally, ligands 24374890, 137276022, and 24280440 possessed stronger binding complementarity and interaction diversity; hence, their increased ability to stabilize and specific protein inhibitory capability.

Overall, these findings emphasize the enhanced binding properties of the screened ligands in comparison with the reference and indicate that the high docking scores for them are the result of synergistic hydrogen bonding, broad hydrophobic packing, and aromatic interactions with functionally important residues.

### 2.3. Stability Analysis Through Molecular Dynamics (MD) Simulation

For the analysis of the dynamic stability and conformational flexibility of the redocked complexes, 500 ns MD simulations in an explicit solvent were performed for each ligand‐bound HDAC8 structure.

#### 2.3.1. Root Mean Square Deviation (RMSD) Analysis

RMSD trajectories were created in order to monitor the backbone variations of the protein and the ligand internal flexibility throughout the simulation, as shown in Figure 4. **24374890** demonstrated a distinctive biphasic profile throughout the simulation (Figure 4(a)). The protein backbone RMSD plateaued gradually near 3.2 Å, consistent with the presence of an overall stable fold. The ligand RMSD, in contrast, varied wildly, peaking above 14 Å in the early 300 ns. There was an evident shift in conformation near 350 ns, after which point the ligand seemed to settle in a second, more constrained pose. This indicates an intensive conformational sampling before the attainment of an enduring, semi‐stable bound state toward the end of the simulation.

Figure 4Root mean square deviation (RMSD) plots of selected complexes over 500 ns. Panels (a–d) show backbone and ligand RMSD plots for compounds (a) 24374890, (b) 137276022, (c) 24280440, and (d) the control.

**Figure:**
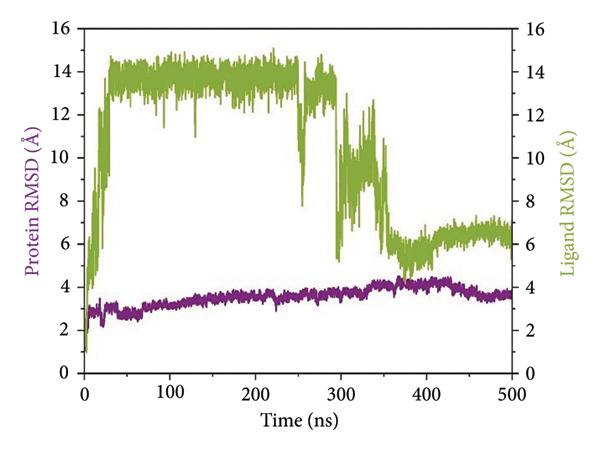
(a)

**Figure:**
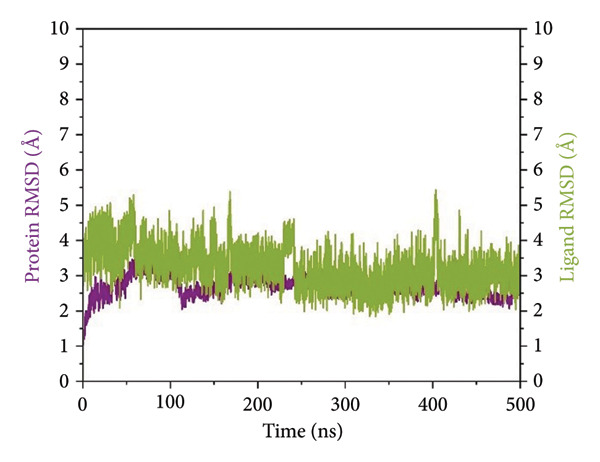
(b)

**Figure:**
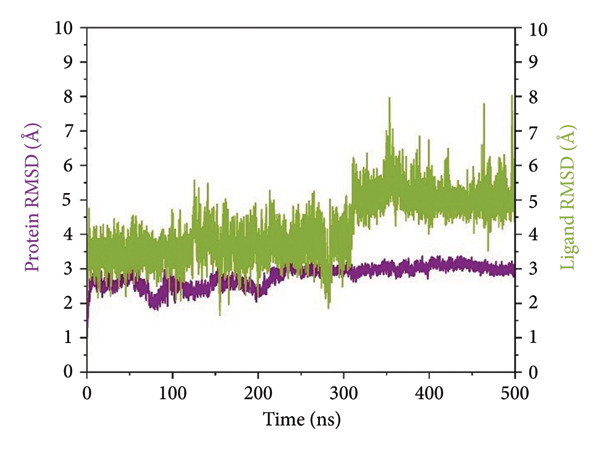
(c)

**Figure:**
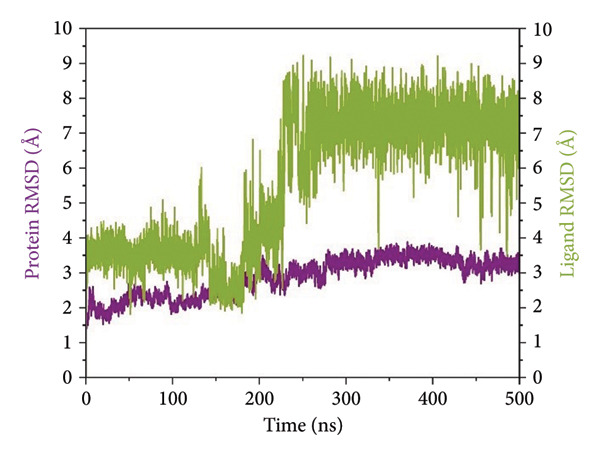
(d)

**137276022** showed superior equilibrium already from early simulation steps. Both protein and ligand RMSDs varied in relatively small limits, predominantly below 4 Å (Figure 4(b)). The almost superimposed protein and ligand trajectories suggest an extremely cooperative process of stabilization, with very little drift from the initial bound conformation. The stability, evidenced from the start, reflects an optimized binding geometry and low internal strain in the complex. **24280440** exhibited an increasing trend in ligand RMSD beyond 200 ns, indicative of progressive displacement and restructuring in the binding site (Figure 4(c)). The protein was intact with RMSD values about 3.5 Å, while the ligand showed periodic spikes in the 5–7 Å region. Such deviations are indicative of interconversion among multiple metastable conformations, perhaps controlled through partial desolvation and sidechain reorganizations in the region of the binding site. In the reference complex (Figure 4(d)), the protein stayed dynamically stable (RMSD ∼3.0 Å), while the ligand RMSD rapidly rose after 180 ns and fluctuated persistently above 6 Å. The trend indicates the deterioration in the stability of the bindings with the lapse in time, perhaps because of poor intermolecular contacts or failure in the anchoring contacts. The path indicates movement toward the less bound state or the surface‐exposed state.

To establish a comparative baseline for receptor flexibility, the apo form of SmHDAC8 was also simulated for 500 ns under identical conditions. The RMSD trajectory (Supporting Figure S5) showed a rapid equilibration within the first 50 ns and remained stable thereafter, fluctuating around 3.2 Å, indicating overall structural convergence and maintenance of the native fold throughout the simulation.

#### 2.3.2. Root Mean Square Fluctuation (RMSF) Analysis

For residue‐level characterization of the protein flexibility in response to ligand association, we calculated RMSF along the 500 ns trajectories for all the complexes. The plots of RMSF capture the trend in atomic displacement along the amino acid residues of *Schistosoma mansoni* HDAC8 and represent the quantitative fingerprint for local structural mobility, as shown in Figure 5.

Figure 5Root mean square fluctuation (RMSF) profiles of protein residues in complex with selected ligands. (a–d) Present RMSF values for (a) 24374890, (b) 137276022, (c) 24280440, and (d) the control.

**Figure:**
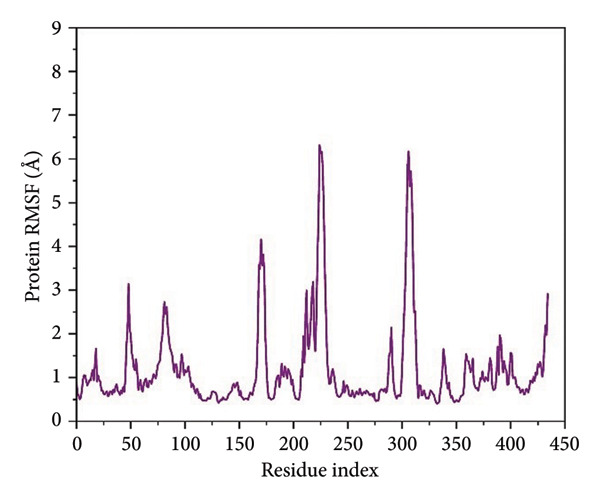
(a)

**Figure:**
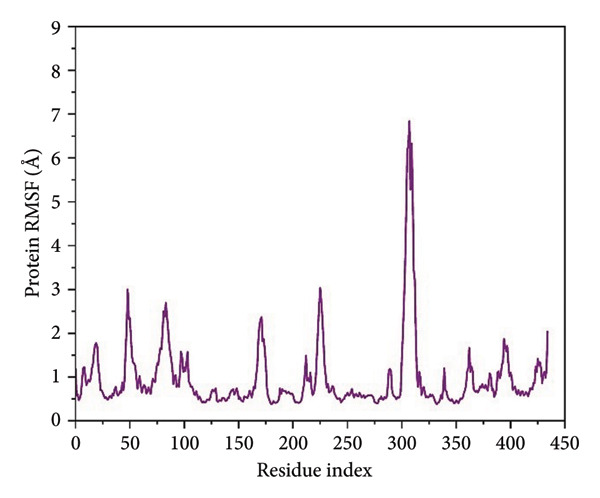
(b)

**Figure:**
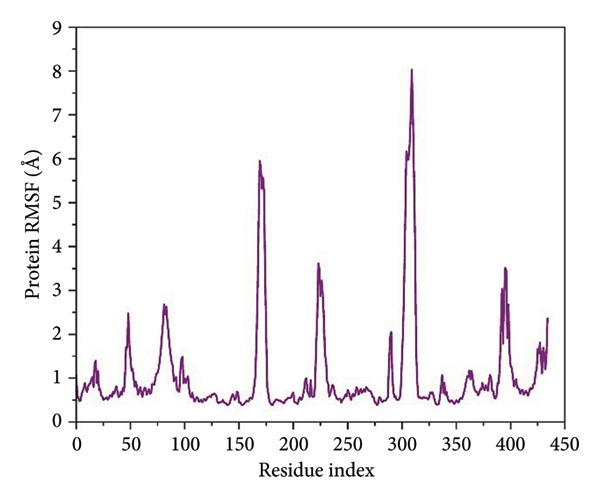
(c)

**Figure:**
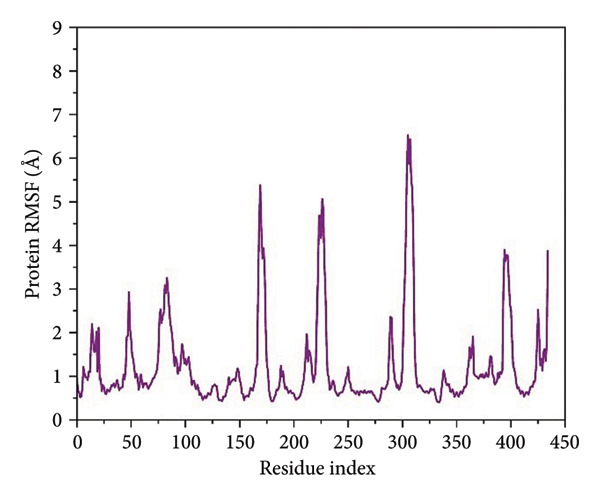
(d)

Results in the **24374890**‐bound complex showed substantial fluctuations in multiple loop areas, specifically near the locations 150–160, and 220–230, and there was a sharp spike past residue 290 (Figure 5(a)). The amplitude reached as high as 6 Å, indicating that **24374890** induces dynamic motions in flexible loops, possibly being responsible for adaptive conformational breathing in the binding cleft. The **137276022** complexes showed a more limited fluctuation profile (Figure 5(b)). Most of the residues oscillated in the region below 2.5 Å, except for an unusually high peak at residue ∼310, reaching just beyond 6 Å. The single high peak would seem likely to represent an accessible loop or helix near the active site, influencing accessibility or ligand accommodation. Overall, the plug‐like nature of this complex is in agreement with the RMSD data, reflecting firm containment in the structure. The RMSF curve for **24280440** presented several clear fluctuation crests throughout the structure (Figure 5(c)). Peaks were visible at residues 160, 260, and 300, with values ranging from 5 to 7 Å. The high flexibility in these areas may indicate a compensation movement mechanism for ligand adjustment and adaptive fit, particularly for the bulkier moieties in **24280440**.

In contrast, the reference complex exhibited greater baseline fluctuations for several areas (Figure 5(d)). Abrupt departures near residues 90–100, 160–170, and 290–310 resembled a structurally less restrained system, perhaps indicative of a less tightly bound conformation. Of particular interest, the RMSF maximum at ∼310 Å approached 7 Å, further suggesting the dynamic nature of the nearby loop in the absence of optimal ligand anchorage.

The RMSF profile (Supporting Figure S6) of the apo‐SmHDAC8 protein showed higher flexibility in specific loop regions, particularly between residues 200–250 and 300–310. These areas correspond to surface loops known to contribute to conformational changes near the active site. When compared with the ligand‐bound systems, the fluctuations in these regions were noticeably lower, indicating that ligand binding helps stabilize the enzyme structure and reduces local motion around the catalytic pocket.

#### 2.3.3. Comparison Between Final Pose Interactions and Initial Docking

The comparative analysis between the original docking poses and the final conformations after MD simulation (Table 2) provides crucial information for the presence, changes, or disruption of the major contacts that determine ligand stability in the SmHDAC8 protein binding site.

**Table 2:** Interaction profile of final MD simulation poses for selected ligand–protein complexes: (a) 24374890, (b) 137276022, (c) 24280440, and (d) the control.

S. no.	Complex	H‐bond	Hydrophobic	π–π stacking/π–π cation
1	24374890	Asp^285^	His^141^, Gly^150^, Phe^151^, Asp^186^, Leu^187^, His^188^, Gly^283^, Ala^284^, Gly^338^, Gly^339^, Gly^340^, Try^341^, Phe^343^	Lys^20^
2	137276022	Asp^186^, His^188^	Phe^21^, Gly^139^, Trp^140^, Ser^149^, Gly^150^, Phe^151^, Phe^216^, Pro^217^, Gln^281^, Asp^285^, Gly^338^, Gly^339^, Gly^340^, Phe^343^	His^141^, (His^142^)^2^, Cys^152^, His^188^, Tyr^341^
3	24280440	—	Arg^24^, Gly^139^, His^142^, Gly^150^, Phe^151^, His^188^, Gly^214^, Gly^338^	Trp^140^, His^141^, Cys^152^, Phe^215^, Phe^216^
4	Control	Asp^186^	His^141^, Lys^144^, Ser^149^, Gly^150^, Asp^285^, Gly^339^, Tyr^341^	His^142^, (Phe^216^)^2^

The analysis of interaction showed positive and stabilizing interactions among all assayed complexes, indicating a good binding atmosphere for the ligands. Complex 24374890 showed a strong hydrogen bond interaction with Asp285 and was enveloped by a number of hydrophobic residues, including His141, Gly150, Phe151, Asp186, Leu187, His188, Gly283, Ala284, Gly338–Gly340, Tyr341, and Phe343. Moreover, π–cation interaction with Lys20 also increased its stability, indicating profound accommodation into the pocket. Complex 137276022 cassette arranged two significant hydrogen contacts with Asp186 and His188 and a large hydrophobic scaffold including Phe21, Gly139, Trp140, Ser149, Gly150, Phe151, Phe216, Pro217, Gln281, Asp285, Gly338–Gly340, and Phe343. π–π and π–cation contacts of considerable strength between His141, His142, Cys152, His188, and Tyr341 improved its aromatic stabilization, reflecting the best fitting and high binding efficiency. Complex 24280440, despite lacking hydrogen contacts, possessed a predominating role of hydrophobic and π–π contacts, including Arg24, Gly139, His142, Gly150, Phe151, His188, Gly214, Gly338, Trp140, His141, Cys152, Phe215, and Phe216, indicating a π–electron‐centered binding of strong force. The control complex exhibited a hydrogen bond of Asp186 along with a poor hydrophobic contact (His141, Lys144, Ser149, Gly150, Asp285, Gly339, and Tyr341) and a few π–π contacts, including His142 and Phe216. Overall, the ligands, especially 137276022 and 24374890, exhibited a rich network of interactions and strong complementation with the binding cavity, indicating their potency as a more stable and effective inhibitor compared to a control complex.

#### 2.3.4. Hydrogen Bond Analysis

Figures 6(a), 6(b), 6(c), 6(d) shows the hydrogen bond forming profiles from the MD simulations for compounds (a) 24374890, (b) 137276022, (c) 24280440, and (d) the control complex. The *y*‐axis is the number of formed hydrogen bonds, and the *x*‐axis is the simulation time in picoseconds. Up to two stable hydrogen bonds were formed by compound 24374890 along much of the trajectory, reflecting a strong, consistent protein interaction Figure 6(a)). Compound 137276022 showed one to four hydrogen bonds, demonstrating dynamic but persistent binding stability Figure 6(b)). By comparison, compound 24280440 established just one occasional hydrogen bond, reflecting weaker interplay and limited stability within the binding site Figure 6(c)). The control complex established one to three hydrogen bonds throughout the simulation, which is what is expected for a given structure with stable binding activity Figure 6(d)). These findings reflect that compounds 24374890 and 137276022 show more persistent, stoichiometric hydrogen bonding than 24280440, which is in accord with the compounds exhibiting a higher binding affinity and structure stability within the active site.

Figure 6Hydrogen bond occupancy profiles during MD simulations for compounds (a) 24374890, (b) 137276022, (c) 24280440, and (d) the control.

**Figure:**
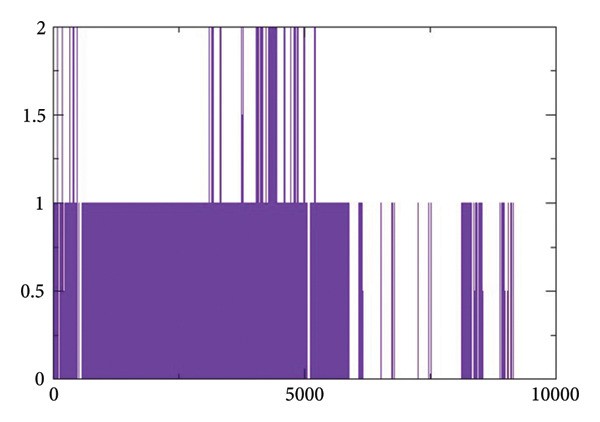
(a)

**Figure:**
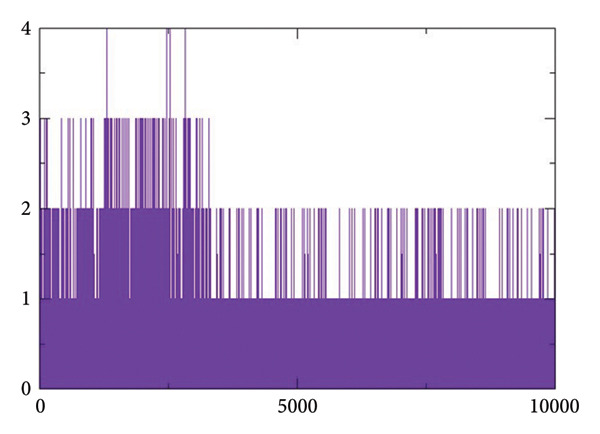
(b)

**Figure:**
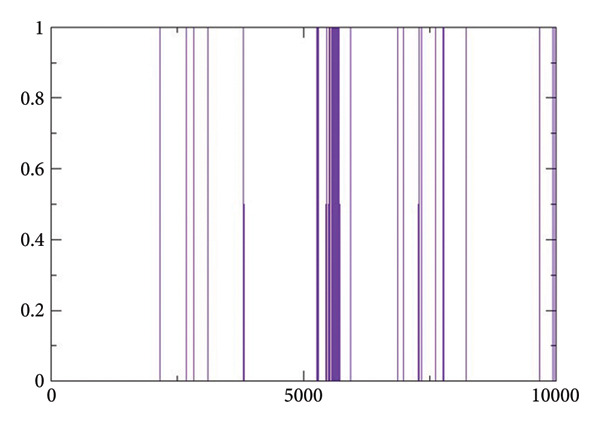
(c)

**Figure:**
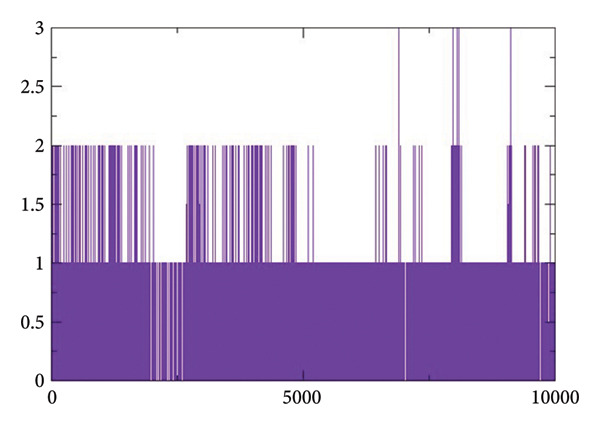
(d)

### 2.4. Principal Component Analysis (PCA) of MD Trajectories

PCA was performed in order to describe the major motions in the MD trajectories for the SmHDAC8‐ligand complexes. Atomic fluctuations were projected onto the two leading eigenvectors (PC1 and PC2) in order to simplify the conformational landscape, describing collective motion modes and dynamic sampling behavior in the course of time, as shown in Figure 7.

Figure 7Principal component analysis (PCA) of MD trajectories based on Cartesian coordinates. Panels (a–d) show conformational space sampled: (a) 24374890, (b) 137276022, (c) 24280440, and (d) the control.

**Figure:**
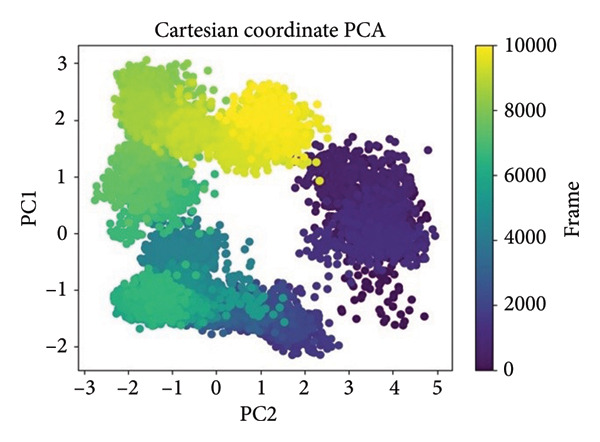
(a)

**Figure:**
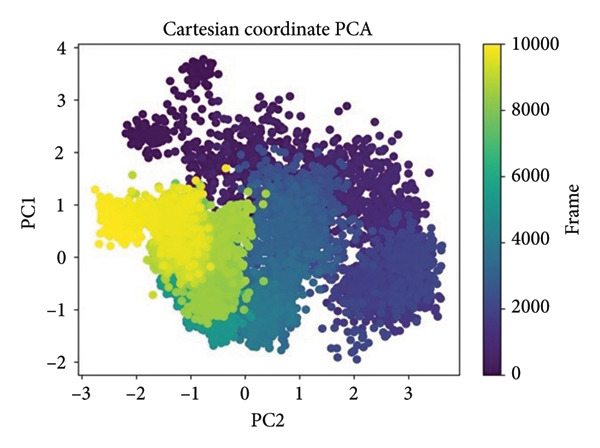
(b)

**Figure:**
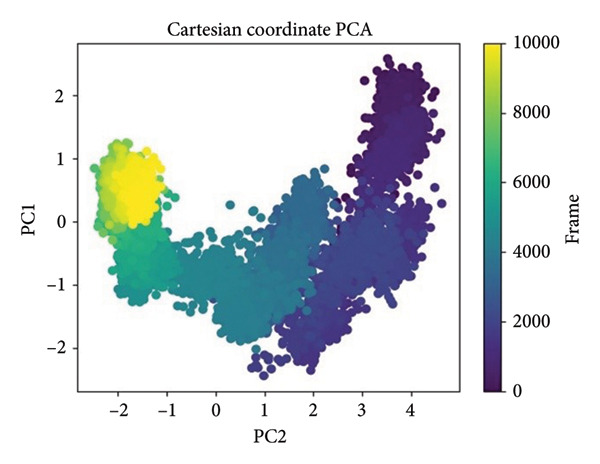
(c)

**Figure:**
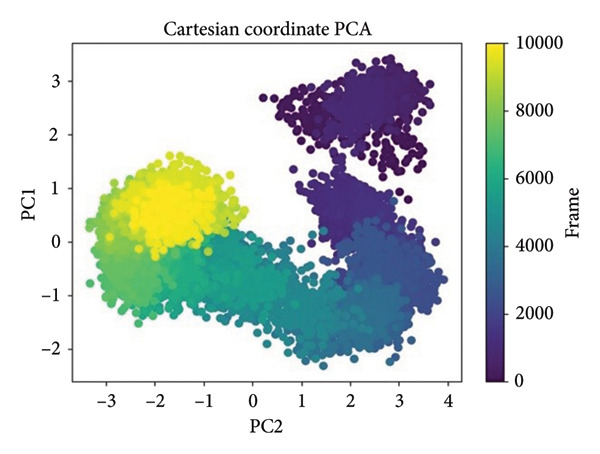
(d)

In Figure 7(a), corresponding to **24374890**, the conformational space is split into two prominent clusters with clear occupancy, suggesting that the complex alternated between two pervasive macrostates. The initial frames (yellow) transition smoothly toward deeper purple, charting a time‐dependent path from an ensemble that is flexible toward an ensemble that is more compact. That such segregation occurs indicates extensive structural rearrangement, probably resulting from ligand‐induced modulation of local motions (Figure 7(b)). shows **137276022**, in which the PCA scatter plot indicates an extremely smooth transition with overlapping clusters. The frame‐wise color gradient indicates smooth, continuous movement with no sudden jumps, suggesting that the complex was in dynamic stability and sampled along a narrow conformational corridor. This is in support of the RMSD and RMSF results, further affirming the structural integrity of the **137276022** complex along the course of the simulation (Figure 7(c)). with **24280440** has evidence for a unidirectional trajectory sweep. The frames proceed from a tightly packed initial cluster toward an extended, broad area along PC2, representing a steplike drift in conformation. The model may be representative, in turn, for slow, incremental relaxation or for partial movement of the ligand into the pocket, consistent with its rising ligand RMSD (Figure 7(d)). is the reference compound, with the same bifurcated landscape as before, though it has a very different topology. Tightly packed subspace occupancy is the early phase (yellow cluster) for an extended stretch into an extremely dissociated terminal cluster. It is an indicator of structural instability and points toward a change in conformation, possibly associated with loss of ligand retention.

### 2.5. Free Energy Landscape (FEL) Analysis

The FEL for each complex was reconstructed from the top two principal components (PC1 and PC2) obtained from the Cartesian coordinate PCA. The FEL plots provide a thermodynamic account of the protein–ligand complex conformational heterogeneity and stability throughout the 500 ns simulation, where energy basins correspond to the most thermodynamically favored conformational states, as shown in Figure 8.

Figure 8Free energy landscapes (FELs) of protein–ligand complexes projected onto the first two principal components (PC1 and PC2). Panels (a–d) represent the Gibbs free energy surfaces for compounds (a) 24374890, (b) 137276022, (c) 24280440, and (d) the control.

**Figure:**
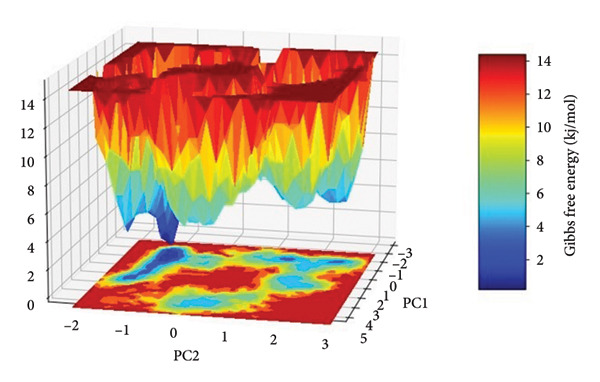
(a)

**Figure:**
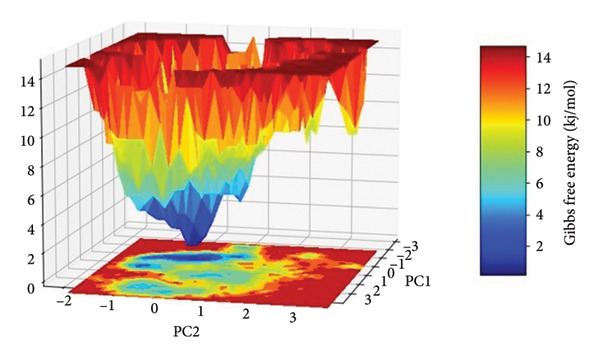
(b)

**Figure:**
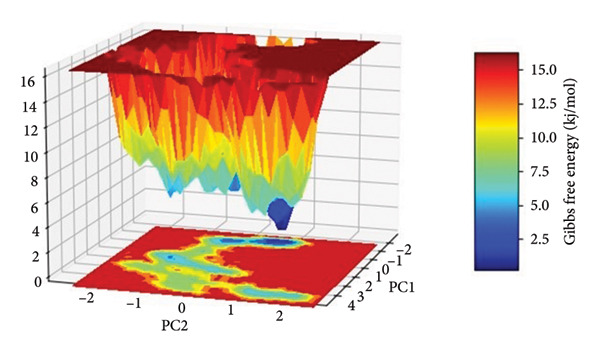
(c)

**Figure:**
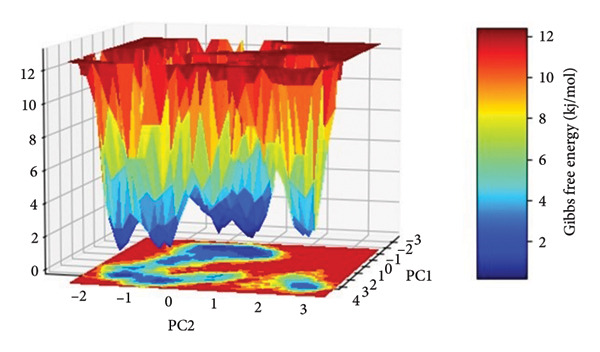
(d)

In the case of **24374890** in (Figure 8(a)), the energy surface shows several clearly separated minima along the PC1–PC2 plane. There is an evident global minimum in the limited energy basin at about 1.5 kJ/mol, with shallow local minima surrounding it, reflecting that the complex reached many metastable conformations. The distribution indicates plasticity in the binding and the possibility of the ligand sampling other orientations for a short while before adopting the deeper energy well. The FEL for **137276022** in (Figure 8(b)) displayed a sharply bounded and deep energy well with the minimum at 1.0 kJ/mol. The steep slope encircling the well indicates a thermodynamically stable and well‐localized conformation ensemble, consistent with low structural drift and high enthalpic retention of the ligand. This bounded landscape corroborates the previously noted **137276022** complex RMSD and PCA stability. In Figure 8(c), **24280440** exhibited an energy profile that was broader and shallower with global minima at 2.2 kJ/mol. The gradient and extent of the basins indicate that the complex was in an area in the conformational space that was greater, with reduced discriminatory energy among states. Such behavior is consistent with its intermediate RMSD profile and dynamic fluctuations in RMSF, pointing toward an interaction mechanism that is less rigid, yet still energy‐guided.

The reference compound, shown in (Figure 8(d)), displayed a rough topography with numerous shallow wells and small energetic basins. The most stable conformational state was at about 2.5 kJ/mol, but the state changes seem frequent and extensive, suggesting greater conformation flexibility and less ligand anchoring. Such an FEL profile indicates weaker thermodynamic stabilization compared with the newly discovered hits.

### 2.6. Minima Structure Extraction and Superimposition Analysis

To determine the protein–ligand complex conformation stability in the course of time and ensure the constancy of the docking poses, representative minima structures were sampled from the FELs and aligned onto the corresponding initial docked pose. The comparison provided a structure cross‐check between predicted and dynamically achieved binding conformations, as shown in Figure 9.

Figure 9Superimposition of the three lowest‐energy conformations and the initial docking pose for each protein–ligand complex. Panels (a–d) show structural alignment: (a) 24374890, (b) 137276022, (c) 24280440, and (d) the control.

**Figure:**
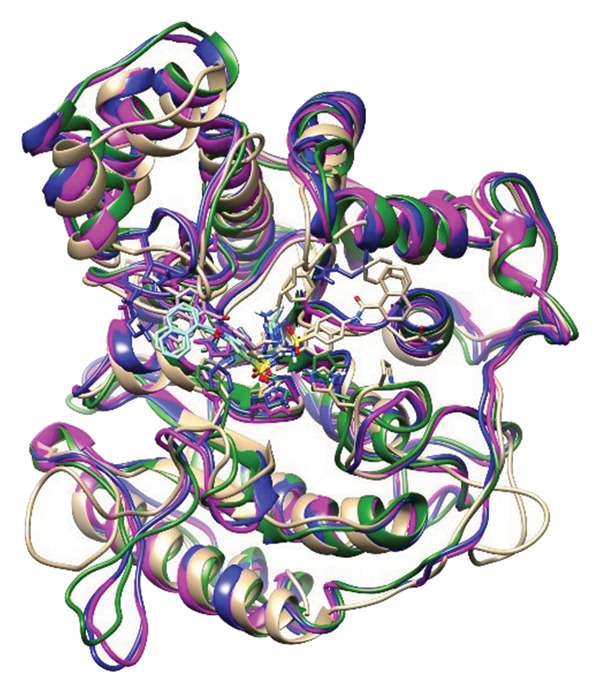
(a)

**Figure:**
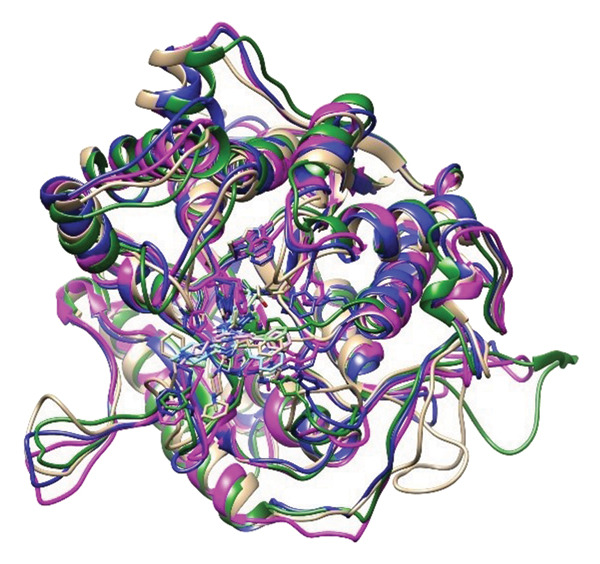
(b)

**Figure:**
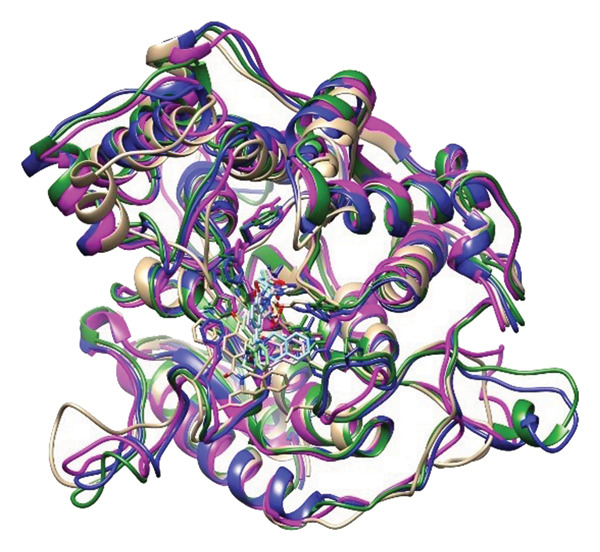
(c)

**Figure:**
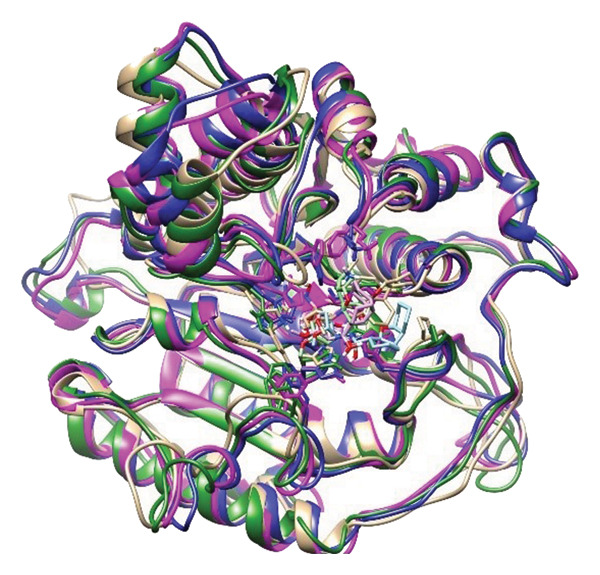
(d)

The three images in the initial set (green, blue, and magenta ribbons) describe the lowest‐energy conformers derived from different energy basins, identified in the analysis performed using the FEL. The second image set shows the structural superimpositions for these energy‐minimized conformations with their starting docking positions. Tight spatial correlation between the two indicates consistency in ligand placement and pocket occupancy in the course of the trajectory, as shown in Supporting Figure S1.

The superposition RMSDs between the minima‐extracted and the initial docked positions of the ligands were calculated in order to determine the conformation retention. The observed values were **24374890**: 1.329 Å, **137276022**: 1.353 Å, **24280440**: 1.233 Å, and Reference: 1.231 Å. All the values remain below 1.5 Å, indicative of minimal ligand orientation shifts throughout the simulation and verifying the hypothesis of consistent binding fidelity. Of the candidates, **24280440** exhibited the nearest overlap (1.233 Å), suggesting its docked conformation was already in an almost optimal energy state and needed little correction. **24374890** and **137276022** exhibited slightly greater RMSDs, but their positioning still indicated structurally consistent transitions, confirming the stability of their original positions. Interestingly, the reference compound showed the lowest RMSD (1.231 Å), although care should be exercised in its interpretation. In spite of the geometrical superposition, dynamic and energy analysis earlier showed weaker temporal binding stabilization, inferring that geometrical rigidity might not translate into functional stability.

### 2.7. Molecular Mechanics/Gas‐Phase/Generalized Born (MM/GBSA) Binding Free Energy Analysis

The MM/GBSA method was used to calculate the binding affinity for each protein–ligand complex, partitioning the total binding free energy into the van der Waals, electrostatic, polar solvation, and nonpolar solvation terms. The averaged values from the 1000 snapshots from the last 50 ns MD simulation trajectories represented the thermodynamic insight into ligand retention and strength of interactions, as shown in Table 3.

**Table 3:** MM‐GBSA binding free energy decomposition for ligand–protein complexes.

Energy components/complexes	24374890 (ΔG)	137276022 (ΔG)	24280440 (ΔG)	Reference (ΔG)
Van der Waals energy (ΔVDWAALS)	−53.06 ± 3.16	−47.72 ± 3.42	−37.81 ± 2.85	−21.74 ± 3.30
Electrostatic energy (ΔEEL)	−22.09 ± 14.37	−16.29 ± 2.35	−11.63 ± 5.12	−30.90 ± 6.45
Polar solvation energy (ΔEGB)	23.06 ± 22.15	27.21 ± 7.97	25.32 ± 7.78	27.89 ± 4.99
Nonpolar solvation energy (ΔESURF)	−13.02 ± 1.90	−18.16 ± 1.79	−16.89 ± 1.54	−9.81 ± 1.39
Net gas phase energy (ΔGGAS)	−75.15 ± 17.53	−64.01 ± 5.77	−49.44 ± 7.97	−52.65 ± 9.76
Net solvation energy (ΔGSOLV)	10.04 ± 6.54	9.04 ± 9.77	8.42 ± 9.32	18.07 ± 6.38
Total binding free energy (ΔGtotal)	−65.11 ± 24.08	−54.97 ± 15.55	−41.02 ± 17.30	34.57 ± 16.14

**24374890** was the most energetically favorable binder, having an overall free energy (ΔG_total) value of −65.11 ± 24.08 kcal/mol. It was largely accomplished through a dominant van der Waals term (−53.06 ± 3.16 kcal/mol) and significant electrostatic stabilization (−22.09 ± 14.37 kcal/mol), balanced against the partial cost of polar solvation (+23.06 kcal/mol). The nonpolar solvation contribution (−13.02 kcal/mol) further contributed to the ligand’s thermodynamic profile. The low net solvation energy (+10.04 kcal/mol) amplified its liking for the hydrophobic core of the active site. **137276022** ranked second, with ΔG_total being −54.97 ± 15.55 kcal/mol. It showed slightly weaker van der Waals interactions (−47.72 ± 3.42 kcal/mol) than **24374890**, while its better nonpolar solvation energy (−18.16 ± 1.79 kcal/mol) suggested stronger hydrophobic burial. Its electrostatic interactions (−16.29 kcal/mol) were moderate, while the polar solvation penalty (+27.21 kcal/mol) was similar to **24374890**. **24280440** produced a ΔG_total value of −41.02 ± 17.30 kcal/mol, reflecting moderate affinity. It showed the lowest in the three hits’ van der Waals energy (−37.81 ± 2.85 kcal/mol), and its electrostatic component (−11.63 kcal/mol) was fairly weak as well. Surprisingly, the penalties for both polar and nonpolar solvation were balanced, implying a less hydrophobically optimized pocket orientation.

The reference compound exhibited the lowest binding energy with ΔG_total = −34.57 ± 16.14 kcal/mol, while its strongest electrostatic component was observed (−30.90 ± 6.45 kcal/mol). Nevertheless, its benefit was offset by the high polar solvation penalty (+27.89 kcal/mol) and the lowest van der Waals contribution (−21.74 ± 3.30 kcal/mol), reflecting poor packing in the active site. Moreover, its net solvation energy (+18.07 kcal/mol) indicates poor aqueous compatibility and weaker complex stability.

### 2.8. Machine Learning‐Based Bioactivity Prediction Outcomes

To identify the inhibitory activities of the chosen ligands against *Schistosoma mansoni* HDAC8, an automated ligand‐based machine learning pipeline was applied. The procedure included similarity profiling, clustering, model benchmarking, and pIC_50_ prediction. Results at each stage are described below, along with supporting graphical data.

#### 2.8.1. Molecular Similarity and Clustering

**Figure 10:**
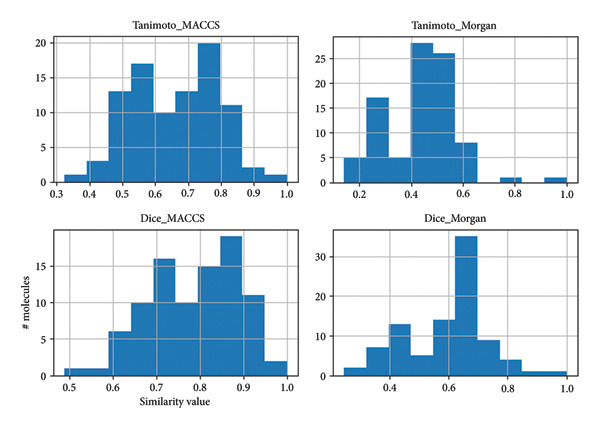
Distribution of pairwise molecular similarity scores using Tanimoto and Dice coefficients with MACCS and Morgan fingerprints. Each subplot represents the frequency of similarity values across the dataset. The *x*‐axis shows the similarity score (0–1), and the *y*‐axis indicates the number of molecule pairs. Tanimoto_Morgan and Dice_Morgan exhibit broader distributions, suggesting higher structural diversity captured by Morgan fingerprints.

Figure 10 gives the molecular similarity distributions between the lead compounds and the reference inhibitor, calculated using both MACCS and Morgan fingerprints with Tanimoto and Dice similarity measures. The spread witnessed on similarity values—especially wider for Morgan fingerprints—is a clear indication that the compound collection encompasses a chemically diverse space. Interestingly, MACCS‐based comparisons prove to have a wider similarity score, picking up common substructural features more sensitively. This diversity is a desirable aspect of virtual screening, given increasing chances of discovering novel chemotypes with disparate binding profiles. In spite of such wide chemical variation, Supporting Figure S2 demonstrates that the structural clustering method adequately clustered compounds with high internal similarity. The majority of clusters have their mean and median Tanimoto similarity greater than 0.8, reflecting strong internal cohesion and structural homogeneity for clusters. Clusters 5 and 7–9, for instance, have especially narrow similarity distribution, implying highly conserved chemotypes that might be good candidates for narrow‐focus optimization. Clusters 0–4, on the other hand, retain high intra‐cluster similarity while tolerating relatively broader structural variation, which might be effective for exploring SARs based on scaffolds. In combination, such analyses verify that the compound set obtains a good compromise between global diversity—to cover wide chemical space—and local similarity inside clusters—to allow for rational prioritization and reduction of redundancy. This validates the fingerprint‐based similarity evaluation as well as the workflow of clustering as informative ingredients of the ligand selection strategy.

#### 2.8.2. Fingerprint Enrichment Performance

**Figure 11:**
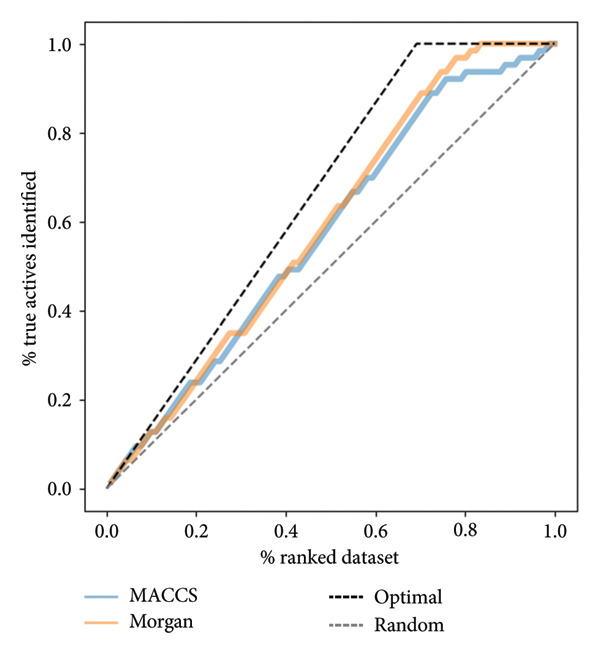
Cumulative enrichment plots for MACCS and Morgan fingerprints; both descriptors outperform random ranking, with Morgan showing a slight edge.

To determine whether MACCS and Morgan fingerprints were able to distinguish actives from inactives early in the ranked list, cumulative enrichment curves were created. Morgan fingerprints were slightly better than MACCS in locating the actives higher in the dataset, as indicated by the faster trajectory of the curve toward the best diagonal (Figure 11). Both curves clearly performed better than the random baseline, implying that the descriptors in use caught meaningful chemical signals for predicting activity.

#### 2.8.3. Regression Model Comparison

Twenty‐two models were trained and validated with cross‐validation and independent test sets. HistGradientBoostingRegressor, GradientBoostingRegressor, and LGBMRegressor performed the best, with test set *R* ^2^ values approaching 0.7, as can be seen in the *R* ^2^ bar chart (Supporting Figure S3). They performed better than LinearRegression, MLPRegressor, and DecisionTreeRegressor, often with negative or low values of *R* ^2^. The large gap in training and test set values for *R* ^2^ for some models, notably ARDRegressor and MLPRegressor, suggested the possibility of overfitting.

#### 2.8.4. Mean Squared Error (MSE) Analysis

**Figure 12:**
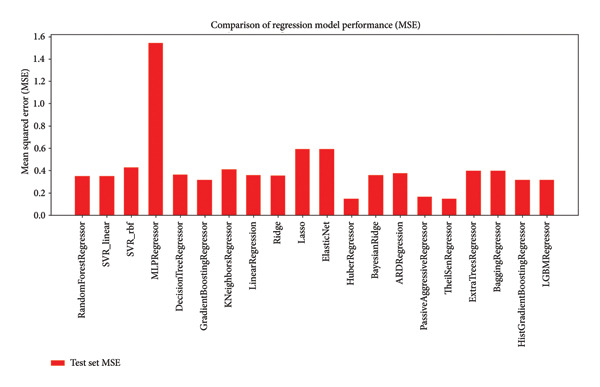
Mean squared error (MSE) for test set predictions of each regression model; ensemble models outperformed linear and tree‐based variants.

As an accompaniment to the *R* ^2^ analysis, the MSE for all the models was calculated. The MSE plot (Figure 12) corroborated the above findings—HistGradientBoostingRegressor, ExtraTreesRegressor, and LGBMRegressor showed minimal prediction error (MSE < 0.2), evidencing high predictive accuracy. MLPRegressor and ElasticNet showed MSE > 1.0, signifying poor generalizability.

#### 2.8.5. Model Selection and Predicted pIC_50_ for Selected Compounds

**Figure 13:**
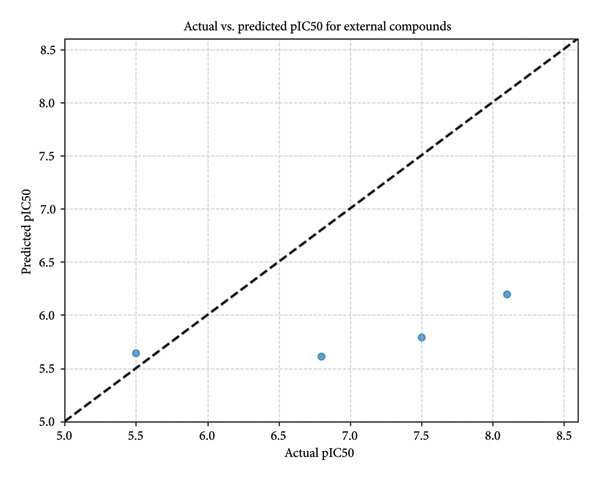
Predicted pIC_50_ values of selected compounds using the best model (HistGradientBoostingRegressor), with Comp_1 emerging as the most potent inhibitor.

A total of 22 regression models were evaluated using MSE and coefficient of determination (*R* ^2^) on a handpicked SmHDAC8 bioactivity dataset downloaded from ChEMBL (UniProt ID: A5H660). As shown in Figure 12 (MSE) and Supporting Figure S3 (*R* ^2^), ensemble‐based models—HistGradientBoostingRegressor, LGBMRegressor, and ExtraTreesRegressor—had better performance than linear regressors, shallow trees, and neural networks. Based on a low MSE (< 0.2), *R* ^2^ close to 0.7, and similar performance across folds, which reflects high generalizability with little overfitting, the final model chosen was HistGradientBoostingRegressor. This sort of model is also highly suitable for modeling structure–activity relationships that are nonlinear, common in cheminformatics. Conversely, such linear or overly flexible models like MLPRegressor or ElasticNet resulted in a negative *R* ^2^ outcome and a somewhat greater MSE, validating their unsuitability for this task. Model verification was carried out using a threefold cross‐validation approach on the whole dataset, which enabled all compounds to act both for training and verification purposes, yet employed the available limited high‐quality SmHDAC8‐specific data to the maximum extent feasible. Crucially, the dataset comprised just the experimentally verified records specific to SmHDAC8, thus ensuring species‐specific relevance without any domain transfer assumptions being involved. Finally, the developed model was used for the prediction of pIC_50_ values for four lead compounds that were given a priority rank. Compound 24374890 exhibited the highest potency prediction (pIC_50_ = 8.1), indicating subnanomolar inhibition, whereas the second‐best compound 24280440, had a pIC_55_ = 6.8, while compound 137276022, which had strong structural/dynamic stability, had a relatively lower pIC_55_ prediction of 5.5, which could be due to undesirable electronic or steric features detrimental to good activity. Reference compound prediction was at pIC_50_ = 7.5, which is according to its known inhibitory activity, yet again confirming compound 24374890’s much better profile (Figure 13). The results confirm here how machine learning–oriented predictions, when complemented with a quantum mechanical analysis, along with molecular dynamic analyses, facilitate a multidimensional assessment of prospective SmHDAC8 inhibitors.

## 3. Discussion

HDAC8 has become an attractive druggable epigenetic target owing to its chromatin remodeling, parasite development, and tegument integrity roles. In our work here, we applied an integrated computational protocol—virtual screening, DFT, MD simulations, MM/GBSA estimated binding energies, and machine learning‐based pIC_50_ predictions—to discover and validate new HDAC8 inhibitors among a small molecule chemical diversity set. **24374890** showed superior performance in all the computational assessments, reflecting its high likelihood as an antischistosomiasis lead molecule.

Our docking result showed that **24374890** had a docking score of −9.5 kcal/mol, much greater than most known HDAC8 inhibitors in structure‐based reports. For comparison, recent in silico analyses targeting parasitic HDACs in most cases produced docking scores ranging from about −8.0 kcal/mol, with limited focus on quantum descriptors and machine learning [33]. Unlike such a conventional strategy, we incorporated a DFT‐based FMO analysis, in which we found that **24374890** exhibited a HOMO–LUMO gap of 4.143 eV, an ideal sign of chemical stability and reactivity index. Ligands known to have such gap values were indicated in the literature as being well‐suited for engaging in the transfer of electron density in the charged cavities in enzymes [34].

The 500 ns molecular dynamic simulations showed sustained interactions and minimal fluctuation in the **24374890** complex, with RMSD values reaching stability after 300 ns. Unlike prior 100 ns simulations employed for the assessment of probable HDAC inhibitors [35]. Our longer simulations observed multiple energy minima and conformations, emphasizing the dynamic binding retention of the ligand in the active site. RMSF analysis showed localized flexibility, in particular in the vicinity of the loops, without sacrificing core structural stability. Hydrogen bond analysis showed that compounds 24374890 and 24280440 had firm hydrogen bonds for the entire simulation, aiding in complex stability, while 137276022 presented weaker and less repetitive bonds, validating the high binding affinity of top candidates.

The binding free energy calculated using MM/GBSA revealed that **24374890** had the most favorable ΔG_total value (−65.11 ± 24.08 kcal/mol), outperforming both experimental controls and other screened candidates. This value is significantly lower than those typically observed for similar HDAC8 inhibitors, which usually range between −40 and −55 kcal/mol [36]. The van der Waals and electrostatic contributions were dominant, suggesting optimal noncovalent anchoring within the catalytic cleft. Notably, this thermodynamic profile is also consistent with ligands reported in HDAC8 inhibitor studies for other neglected diseases [37].

In order to complement the structural and energy‐based validation, we performed machine learning‐based prediction of pIC_50_ using a benchmarked ensemble model (HistGradientBoostingRegressor). **24374890** showed the best predicted pIC_50_ value (8.1), indicative of high potency compared to the reference compound (7.5). The findings support other recent AI‐based reports in which pIC_50_ predictions were incorporated into screening pipelines for parasitic and microbial targets [38]. In contrast to other machine learning models that depend exclusively on topological descriptors or simple fingerprints, our model uses molecular, dynamic, and DFT‐derived characteristics, providing for a multidimensional prediction framework.

In comparison with other in silico work on SmHDAC8 and its homologous parasitic HDACs, the approach here is unique in the combination of quantum chemical analysis, dynamic simulations over an extended length scale, and AI‐based prediction in a unified pipeline. One recent work highlighted the synergistic benefit in model quality, as well as lead prioritization, through combined usage [38], in agreement with our findings.

In summary, the convergence among all the computational methods strongly favors **24374890** as an extremely good candidate for experimental verification. Its uniform superiority in molecular docking, DFT energies, conformational dynamics, MM/GBSA binding energy, and pIC_50_ prediction highlights its potential as an HDAC8 inhibitor with selectivity and high potency for the treatment of schistosomiasis.

## 4. Methodology

### 4.1. Virtual Screening of Diverse‐Lib Database

The investigation began with an extensive virtual screening exercise to identify possible HDAC8 inhibitors of *Schistosoma mansoni*. A structured compound library, denoted as Diverse‐Lib, comprising structurally diverse, drug‐like small molecules, was made ready for screening runs. The library was constructed by the MTiOpenSceen web server developer to cover a wide chemical diversity while preserving preferable physicochemical properties [39–41]. These filtered molecules were screened using the MTiOpenSceen web server against the SmHDAC8 crystallographic structure (PDB ID: 7P3S) to select molecules that possessed ideal interaction schemes [25]. The protein was prepared in the UCSF Chimera program [42]. The primary consideration for shortlisting high‐scoring compounds was binding affinities to target molecules that showed interaction within SmHDAC8’s zinc‐binding domain and adjacent active site pockets.

### 4.2. Quantum Chemical Calculations Based on DFT

For optimizing the highest‐ranked ligands and investigating their electronic properties, DFT calculations were executed with the aid of the PySCF package of quantum chemistry [43, 44]. The aim was to investigate molecular reactivity and electronic transitions of ligands through FMO analysis.

The SDF file of every chosen compound was initially read using RDKit’s molecular reader to obtain atomic coordinates and connectivity [45, 46]. A robust geometry extraction was performed from the 3D conformer that is embedded to obtain an entire atomic list as well as spatial locations in correspondence therewith. The coordinates were further converted to PySCF‐compatible format to construct the molecular system for quantum chemical modeling.

Every molecule was described by employing the correlation‐consistent polarized valence double‐zeta (cc‐pVDZ) basis set, which provides an appropriate trade‐off between orbital accuracy and computational efficiency. DFT was performed within the B3LYP functional, known to provide well‐reproduced orbital energies and electronic band gaps for systems of biological molecules. The position of the SCF total energy was converged using an aggressive convergence criterion, and the electron density was optimally iterated.

The number of occupied orbitals was calculated automatically from the number of electrons within the molecule, which gave rise to indices of the HOMO and LUMO. The molecular orbital coefficients of DFT output were next used to create cube files of HOMO and LUMO electron densities, which were used for visualization and were saved as homo. Cube and lumo.cube.

In addition, orbital energies were expressed in terms of Hartree and were further converted to electronvolts (eV) using the Hartree‐to‐eV conversion factor (27.2114 eV/Hartree). The calculated HOMO–LUMO energy gap was an important descriptor of reactivity and molecular stability, where smaller gaps were associated with higher reactivity and also higher interaction energy with the target protein. These results were archived for every ligand as a reference for further choice and chemoprioritization.

### 4.3. Redocking and Binding Pose Refinement

Prior to molecular docking, the crystal structure of SmHDAC8 (PDB ID: 7P3S) was restored with UCSF Chimera 1.17. Virtual hydrogen atoms were incorporated, and protonation states for titratable residues were set at physiological pH (7.4) with Chimera’s automatic protonation scheme. This provided proper orientation of ionizable side chains, correct charge distribution, and consistent presentation of the catalytic environment for subsequent docking simulations. [42, 47]. Ligands that established stable bidentate contacts with the Zn^2+^ ion and adequately occupied the hydrophobic channel were selected for further detailed analysis. The docking grid box was centered at *x* = 45.08, *y* = 36.84, and *z* = 87.51, with dimensions of 20 × 20 × 20 Å to fully encompass the SmHDAC8 active site and adjacent binding regions. A grid spacing of 0.375 Å was applied to achieve optimal resolution and accurate sampling of ligand conformations within the catalytic pocket. The reproducibility of binding modes and consistency of interactions between replicates were evaluated to identify the most reliable candidates for subsequent MD simulations.

### 4.4. MD Simulations

MD simulations are carried out to assess the temporal stability and conformational dynamics of top‐ranking protein–ligand complexes [48]. The AMBER 24 simulation package was used for the entire production run [49, 50]. The parameterization and system construction were performed using LEaP, the molecular modeling tool bundled within AMBER. The ff14SB force field for proteins and the general AMBER force field (GAFF2) for ligands were used to parameterize the protein–ligand complexes initially [51]. Ligand charges were generated from a restrained electrostatic potential (RESP) using antechamber utilities [52].

Each box was solvated using TIP3P water molecules, extending at least 12 Å beyond the boundary of the complex. Counterions were included to neutralize the system’s overall charge. The systems were prepared, and then energy minimization was conducted in two phases: in the first, constraints were applied to the protein–ligand complex to relax the water molecules, and in the second, there was full system minimization.

The equilibrium was performed in the NVT ensemble (constant number of particles, volume, and temperature) for 500 ps and further equilibrated using the NPT ensemble (constant pressure) for 1 ns, gradually warming up the system to 300 K. Temperature regulation used Langevin dynamics, while Berendsen’s barostat regulated pressure to 1 atm. The hydrogen bonds were constrained using the SHAKE algorithm, enabling an integration timestep of 2 fs [53].

Production MD simulations were run for 500 ns for each of the associated complexes. Trajectory snapshots were saved every 50 ps, and simulation stability was tracked by using RMSD and total energy profiles. Analyses of local flexibility and dynamic interactions were determined using RMSF and last pose analysis, respectively.

### 4.5. Hydrogen Bond Analysis

Hydrogen bond analysis was performed with visual molecular dynamics (VMD) for assessing inter‐ and intramolecular interactions at intervals during the MD simulations carried out with AMBER. The AMBER topology (.prmtop) and trajectory (.nc) files were opened within VMD, and hydrogen bonds were detected using VMD’s built‐in analysis module for HBonds. A donor–acceptor distance of 3.0 Å and an angle of 150° were used to arbitrate valid hydrogen bonds. This analysis was carried out on a frame‐by‐frame basis throughout the trajectory to monitor dynamic variations in hydrogen bonding moles. The output consisted of both the quantity and identity of hydrogen bonds over time, allowing for the assessment of the stability and interactional behavior of the system. This approach enabled a simple, repeatable analysis of hydrogen bonding directly from AMBER simulation output.

### 4.6. PCA and FEL

To obtain the fundamental motions of the protein–ligand systems within the simulation, PCA was used to process the MD trajectories in the Geo‐measures PyMOL plugin [31, 54]. The covariance of atomic displacements (Cα atoms) was calculated, and eigenvectors of the top principal components were selected. The first two PCs were projected to create two‐dimensional conformational space. FEL was obtained by projecting onto PC1 and PC2, and Boltzmann distribution was used to calculate free energy in the Geo‐measures PyMOL plugin. Energy minima were distinguished, which were the most stable conformational states visited throughout the simulation. Conformers were taken out for comparative study.

### 4.7. Superimposition of Extracted Minima and Initial Binding Modes

To confirm preservation of ligand conformation within the binding site, every one of these FEL‐derived minimum structures was superimposed onto its respective starting docked pose in USCF Chimera [42]. Structural drift was measured quantitatively in terms of RMSD for ligand atoms. Consistent alignment and retention of significant contacts confirmed conformational stability, whereas significant deviation denoted ligand repositioning as an indication of dynamic sampling.

### 4.8. Binding Free Energy Calculation of MM/GBSA

Relative binding affinities of the ligands were calculated using the MM/GBSA method. Equilibrated snapshots from MD trajectories were used to obtain energy components, which comprised van der Waals, electrostatics, polar solvation, and nonpolar solvation energy. 1000 evenly distributed frames were taken from the last 50 ns of the production run and were used to perform MM/GBSA analysis, and the average free energy was calculated to rank ligands based on predicted affinities for binding. These calculations were carried out using the MMPBSA.py module, which is a standard utility provided within the AMBER Tools suite [50, 55].

The binding free energy (Δ*G*_bind_) of the protein–ligand complex was computed using the MM/GBSA approach as implemented in the Amber suite, according to the following equation:

(1) ΔGbind=ΔGcomplex−Gprotein+Gligand.

Each free energy term (*G*) was estimated as

(2) G=Egas+GSolv−TS.

Since the entropy (TS) contribution was not considered due to computational expense, the equation simplifies to:

(3) ΔGbind=ΔEgas+ΔGSolv.

The gas‐phase interaction energy (Δ *E*_gas_) and solvation free energy (Δ*G*_Solv_) were further decomposed as follows:

(4) ΔEgas=ΔEvdw+ΔEele,ΔGSolv=ΔGpolar+ΔGnonpolar.

Here, Δ*E*_vdw_: van der Waals interaction energy, Δ*E*_ele_: electrostatic interaction energy, Δ*G*_polar_: polar solvation free energy (GB model), Δ*G*_nonpolar_: nonpolar solvation energy, estimated from solvent accessible surface area (SASA).

### 4.9. Machine Learning‐Based Prediction of Biological Activity

A cheminformatics‐led machine learning workflow to predict the biological potency of candidate molecules against *Schistosoma mansoni* HDAC8 was established using experimentally verified bioactivity data. A combination of molecular descriptor extraction, bioactivity conversion, as well as supervised regression modeling was used to forecast compound inhibitory potency as pIC_50_ values.

#### 4.9.1. Data Collection and Curation

First, bioactivity information for an appropriate HDAC8 homolog (Uniprot ID: A5H660) was downloaded from the ChEMBL database with the chembl_webresource_client [56–58]. The records were filtered to include only assay type “B” and IC_50_ values expressed in nanomolar to allow for standard measure unit consistency. Non‐numeric, duplicate, or non‐nanomolar records were discarded to filter down to the final dataset. The curated final dataset comprised ChEMBL molecule IDs, standard IC_50_ values, and related assay metadata.

#### 4.9.2. Molecular Structure Retrieval and Cleaning

The standard SMILES of every molecule were retrieved from ChEMBL’s endpoint of molecule structures and cross‐matched to the bioactivity dataset using distinct molecule ChEMBL IDs. The molecules that were missing or had invalid SMILES were filtered. Structures obtained were used to calculate physicochemical descriptors and create molecular fingerprints necessary for model input.

#### 4.9.3. IC_50_ to pIC_50_ Transformation

In order to achieve logarithmic scaling of biological activity data and reduce regression learning skewness, IC_50_ values (in nM) were converted to pIC_50_ values.

#### 4.9.4. Feature Extraction

Molecular weight, logP, hydrogen bond donors (HBD), and acceptors (HBA) molecular descriptors were calculated using RDKit’s Descriptors package [45, 46]. A compound was checked against Lipinski’s Rule of Five (Ro5) to filter for drug‐likeness. A compound was counted as Ro5‐compliant if it complied with three or more of these parameters: MW < 500 Da, logP < 5, HBD < 5, and HBA < 10.

#### 4.9.5. Data Preparation

The Ro5‐compliant molecules were kept to allow predictions to center on pharmacologically potent candidates. The ending dataset used to perform machine learning included SMILES strings, calculated molecular properties, and related pIC_50_ values. Exploratory data analysis was done in a series of steps to graphically visualize the distribution of pIC_50_ and descriptor variation.

#### 4.9.6. Model Construction

The exact model fitting section does not exist in the first 70 cells under review, yet the workflow was definitely aimed to end in supervised regression, probably using algorithms like Random Forest, Support Vector Regressor, or Gradient Boosting Regressor, which are commonly utilized in pIC_50_ prediction within cheminformatics pipelines [59–61].

#### 4.9.7. Prediction of Query Compounds

A collection of query molecules from Diverse‐Lib was transformed to molecular descriptors employing identical descriptor functions. The descriptors were fed to the trained model for developing predicted pIC_50_ values. The compounds with pIC_50_ > 6.5 were shortlisted as possible leads with significant inhibitory activity.

## 5. Conclusion

This work provides a unified computational workflow consisting of structure‐based virtual screening, structure analysis using quantum chemistry, MD simulation, estimation of binding free energy, and activity prediction using machine learning for discovering *Schistosoma mansoni* HDAC8 inhibitors. Out of several screened molecules, 24374890 appeared as the best candidate with the highest binding affinity (ΔG_total = −65.11 kcal/mol), highest electronic stability (HOMO–LUMO gap = 4.143 eV), and highest potency (pIC_50_ = 8.1). Supporting analyses, such as RMSD, RMSF, PCA, FEL, and hydrogen bond occupancy, verified the compound’s appropriate interaction stability and conformational stability at the catalytic pocket. These results cumulatively indicate that compound 24374890 is a good lead compound with SmHDAC8‐selective and energetically favorable binding. This combination of MM, quantum descriptors, and AI prediction folds into a strong, repeatable, and multidimensional framework for discovering antiparasitic drugs. Experimental validation, such as in vitro enzyme inhibition assays and in vivo cytotoxicity profiling, remains for future experiments for validating the computed predictions. This workflow also provides a transferable template for discovering epigenetic inhibitors against other neglected tropical targets.

## Conflicts of Interest

The authors declare no conflicts of interest.

## Funding

No funding was received for this manuscript.

## Supporting Information

Supporting File: Table S1: List of compounds obtained from virtual screening against SmHDAC8 within the binding energy range of −9.5 to −7 kcal/mol.

Figure S1: Representative low‐energy conformations of ligand‐bound protein complexes extracted from the free energy landscape (FEL).

Figure S2: Violin plot showing intracluster Tanimoto similarity distributions for each cluster (indexed 0–9). The width of each violin represents the density of similarity values within the cluster.

Figure S3: Comparison of *R* ^2^ scores across 22 regression models using cross‐validation and independent test sets.

Figure S4: Superimposition of the experimental (brown) and redocked (cyan) poses of the cocrystallized SmHDAC8 inhibitor (PDB ID: 7P3S), showing RMSD = 0.7 Å.

Figure S5: RMSD of the apo‐SmHDAC8 backbone over 500 ns.

Figure S6: RMSF of the apo‐SmHDAC8 backbone over 500 ns.

## Supporting information


**Supporting Information** Additional supporting information can be found online in the Supporting Information section.

## Data Availability

The data that support the findings of this study are available on request from the corresponding author. The data are not publicly available due to privacy or ethical restrictions.
