# Supplementary material for: Quantum‐Mechanically Refined, Dynamics‐Coupled, and AI‐Augmented Elucidation of Epigenetic Inhibition: An In Silico Paradigm Targeting HDAC8 of Schistosoma mansoni
Source: J Trop Med. 2025 Dec 22;2025:1172449. doi: 10.1155/jotm/1172449 (PMC12721395; doi:10.1155/jotm/1172449)
Supplement: Supplementary file 1 — Supporting Information Additional supporting information can be found online in the Supporting Information section. [file JOTM-2025-1172449-s001.docx]

| S.no | **Compound** | **Energy** |
| --- | --- | --- |
| 1 | 24374890 | -9.5 |
| 2 | 137276022 | -9.4 |
| 3 | 24280440 | -9.1 |
| 4 | 14720497 | -9 |
| 5 | 858499 | -9 |
| 6 | 99361075 | -8.8 |
| 7 | 4258740 | -8.7 |
| 8 | 843872 | -8.7 |
| 9 | 17504861 | -8.7 |
| 10 | 24268765 | -8.7 |
| 11 | 49732383 | -8.6 |
| 12 | 103060682 | -8.5 |
| 13 | 49828334 | -8.5 |
| 14 | 49828612 | -8.5 |
| 15 | 56319411 | -8.5 |
| 16 | 24343563 | -8.5 |
| 17 | 859812 | -8.5 |
| 18 | 124950699 | -8.4 |
| 19 | 26731901 | -8.4 |
| 20 | 85146752 | -8.4 |
| 21 | 103060169 | -8.4 |
| 22 | 49672022 | -8.4 |
| 23 | 134215354 | -8.3 |
| 24 | 24826015 | -8.3 |
| 25 | 103162789 | -8.3 |
| 26 | 14745763 | -8.3 |
| 27 | 24782867 | -8.3 |
| 28 | 24390277 | -8.3 |
| 29 | 24417279 | -8.3 |
| 30 | 26626190 | -8.3 |
| 31 | 47200504 | -8.3 |
| 32 | 90945003 | -8.2 |
| 33 | 24370788 | -8.2 |
| 34 | 24321689 | -8.2 |
| 35 | 124948790 | -8.2 |
| 36 | 49719376 | -8.2 |
| 37 | 4257416 | -8.2 |
| 38 | 24374992 | -8.2 |
| 39 | 24779968 | -8.2 |
| 40 | 17468957 | -8.2 |
| 41 | 26622256 | -8.2 |
| 42 | 24838666 | -8.1 |
| 43 | 26620144 | -8.1 |
| 44 | 24819698 | -8.1 |
| 45 | 14732732 | -8.1 |
| 46 | 7966478 | -8.1 |
| 47 | 861664 | -8.1 |
| 48 | 26641568 | -8.1 |
| 49 | 24786505 | -8.1 |
| 50 | 24794792 | -8.1 |
| 51 | 85176625 | -8.1 |
| 52 | 26532798 | -8.1 |
| 53 | 17510287 | -8.1 |
| 54 | 14731765 | -8.1 |
| 55 | 24323703 | -8.1 |
| 56 | 24802573 | -8.1 |
| 57 | 49670430 | -8.1 |
| 58 | 24798974 | -8 |
| 59 | 26726071 | -8 |
| 60 | 47200089 | -8 |
| 61 | 24321821 | -8 |
| 62 | 103163258 | -8 |
| 63 | 26651249 | -8 |
| 64 | 26539697 | -8 |
| 65 | 24327210 | -8 |
| 66 | 22406285 | -8 |
| 67 | 24818117 | -8 |
| 68 | 24786926 | -8 |
| 69 | 24828300 | -8 |
| 70 | 24337587 | -8 |
| 71 | 26648312 | -8 |
| 72 | 17515665 | -8 |
| 73 | 47204541 | -8 |
| 74 | 26617391 | -8 |
| 75 | 17462611 | -8 |
| 76 | 50105200 | -8 |
| 77 | 57258192 | -8 |
| 78 | 24317932 | -8 |
| 79 | 49681726 | -8 |
| 80 | 26540072 | -8 |
| 81 | 49816386 | -8 |
| 82 | 49826869 | -8 |
| 83 | 24827181 | -8 |
| 84 | 124949700 | -8 |
| 85 | 26613474 | -8 |
| 86 | 24835182 | -8 |
| 87 | 14728350 | -7.9 |
| 88 | 17476177 | -7.9 |
| 89 | 17450710 | -7.9 |
| 90 | 22416201 | -7.9 |
| 91 | 56373554 | -7.9 |
| 92 | 49640535 | -7.9 |
| 93 | 17459440 | -7.9 |
| 94 | 843513 | -7.9 |
| 95 | 16953535 | -7.9 |
| 96 | 49680935 | -7.9 |
| 97 | 24818736 | -7.9 |
| 98 | 24334593 | -7.9 |
| 99 | 24357126 | -7.9 |
| 100 | 17410443 | -7.9 |
| 101 | 124756730 | -7.9 |
| 102 | 860538 | -7.9 |
| 103 | 4250579 | -7.9 |
| 104 | 85148397 | -7.9 |
| 105 | 26729167 | -7.9 |
| 106 | 56322754 | -7.9 |
| 107 | 17480798 | -7.9 |
| 108 | 26725802 | -7.9 |
| 109 | 57261321 | -7.9 |
| 110 | 26527703 | -7.9 |
| 111 | 846866 | -7.9 |
| 112 | 26618335 | -7.9 |
| 113 | 26661163 | -7.9 |
| 114 | 17481115 | -7.9 |
| 115 | 49819902 | -7.9 |
| 116 | 22407979 | -7.9 |
| 117 | 16953181 | -7.9 |
| 118 | 24822900 | -7.9 |
| 119 | 4259521 | -7.9 |
| 120 | 92764250 | -7.9 |
| 121 | 17454203 | -7.9 |
| 122 | 24342978 | -7.9 |
| 123 | 124948133 | -7.9 |
| 124 | 26539518 | -7.9 |
| 125 | 24806688 | -7.9 |
| 126 | 26541002 | -7.9 |
| 127 | 103075841 | -7.9 |
| 128 | 57259792 | -7.9 |
| 129 | 49644085 | -7.9 |
| 130 | 4260101 | -7.8 |
| 131 | 24399075 | -7.8 |
| 132 | 26731264 | -7.8 |
| 133 | 14723823 | -7.8 |
| 134 | 125305695 | -7.8 |
| 135 | 24837702 | -7.8 |
| 136 | 17455447 | -7.8 |
| 137 | 14725180 | -7.8 |
| 138 | 85270733 | -7.8 |
| 139 | 49718577 | -7.8 |
| 140 | 24799781 | -7.8 |
| 141 | 24282800 | -7.8 |
| 142 | 99456826 | -7.8 |
| 143 | 4265144 | -7.8 |
| 144 | 24361661 | -7.8 |
| 145 | 17517186 | -7.8 |
| 146 | 24805463 | -7.8 |
| 147 | 50086412 | -7.8 |
| 148 | 24356803 | -7.8 |
| 149 | 48410242 | -7.8 |
| 150 | 14734111 | -7.8 |
| 151 | 24796378 | -7.8 |
| 152 | 47200590 | -7.8 |
| 153 | 24304260 | -7.8 |
| 154 | 24800740 | -7.8 |
| 155 | 124894396 | -7.8 |
| 156 | 26669434 | -7.8 |
| 157 | 3715903 | -7.8 |
| 158 | 24307072 | -7.8 |
| 159 | 26728417 | -7.8 |
| 160 | 99359866 | -7.8 |
| 161 | 24800688 | -7.8 |
| 162 | 7977461 | -7.8 |
| 163 | 26726010 | -7.7 |
| 164 | 17461528 | -7.7 |
| 165 | 26658140 | -7.7 |
| 166 | 26642421 | -7.7 |
| 167 | 14723822 | -7.7 |
| 168 | 14741311 | -7.7 |
| 169 | 4263624 | -7.7 |
| 170 | 124949916 | -7.7 |
| 171 | 104224691 | -7.7 |
| 172 | 26646476 | -7.7 |
| 173 | 26543251 | -7.7 |
| 174 | 17471831 | -7.7 |
| 175 | 4257376 | -7.7 |
| 176 | 49721284 | -7.7 |
| 177 | 124349941 | -7.7 |
| 178 | 49731741 | -7.7 |
| 179 | 49647867 | -7.7 |
| 180 | 4245889 | -7.7 |
| 181 | 24320149 | -7.7 |
| 182 | 24836059 | -7.7 |
| 183 | 49728662 | -7.7 |
| 184 | 24786325 | -7.7 |
| 185 | 4244375 | -7.7 |
| 186 | 24786626 | -7.7 |
| 187 | 99431270 | -7.7 |
| 188 | 56316212 | -7.7 |
| 189 | 56373890 | -7.7 |
| 190 | 49733535 | -7.7 |
| 191 | 3711702 | -7.7 |
| 192 | 17407621 | -7.7 |
| 193 | 17401863 | -7.7 |
| 194 | 124948641 | -7.7 |
| 195 | 17449907 | -7.7 |
| 196 | 51088938 | -7.7 |
| 197 | 24405043 | -7.7 |
| 198 | 57261576 | -7.7 |
| 199 | 49727910 | -7.7 |
| 200 | 24826359 | -7.7 |
| 201 | 26725687 | -7.7 |
| 202 | 17412145 | -7.7 |
| 203 | 7971098 | -7.7 |
| 204 | 24351475 | -7.7 |
| 205 | 24833093 | -7.7 |
| 206 | 26649838 | -7.7 |
| 207 | 24833484 | -7.7 |
| 208 | 7964974 | -7.7 |
| 209 | 24331969 | -7.7 |
| 210 | 26641090 | -7.7 |
| 211 | 47196427 | -7.7 |
| 212 | 74373859 | -7.7 |
| 213 | 22412636 | -7.7 |
| 214 | 24779973 | -7.7 |
| 215 | 24322490 | -7.7 |
| 216 | 17408664 | -7.7 |
| 217 | 26725928 | -7.7 |
| 218 | 26647087 | -7.7 |
| 219 | 24399112 | -7.7 |
| 220 | 49824580 | -7.7 |
| 221 | 4262821 | -7.7 |
| 222 | 85269260 | -7.7 |
| 223 | 99495200 | -7.7 |
| 224 | 17504057 | -7.7 |
| 225 | 85267157 | -7.7 |
| 226 | 24824641 | -7.7 |
| 227 | 24794437 | -7.7 |
| 228 | 24291860 | -7.7 |
| 229 | 49720963 | -7.7 |
| 230 | 24292058 | -7.7 |
| 231 | 24280055 | -7.7 |
| 232 | 26617162 | -7.7 |
| 233 | 24372634 | -7.7 |
| 234 | 17442513 | -7.7 |
| 235 | 49732108 | -7.7 |
| 236 | 57265481 | -7.7 |
| 237 | 17408940 | -7.7 |
| 238 | 14721191 | -7.7 |
| 239 | 49736245 | -7.7 |
| 240 | 24825414 | -7.7 |
| 241 | 17446495 | -7.7 |
| 242 | 26642425 | -7.7 |
| 243 | 24790405 | -7.7 |
| 244 | 26728234 | -7.6 |
| 245 | 24334243 | -7.6 |
| 246 | 124948241 | -7.6 |
| 247 | 124898874 | -7.6 |
| 248 | 14723690 | -7.6 |
| 249 | 7967092 | -7.6 |
| 250 | 26617046 | -7.6 |
| 251 | 124948748 | -7.6 |
| 252 | 26648354 | -7.6 |
| 253 | 843985 | -7.6 |
| 254 | 125264920 | -7.6 |
| 255 | 49670135 | -7.6 |
| 256 | 26730904 | -7.6 |
| 257 | 14727639 | -7.6 |
| 258 | 144097705 | -7.6 |
| 259 | 24373719 | -7.6 |
| 260 | 26616765 | -7.6 |
| 261 | 24815672 | -7.6 |
| 262 | 17458112 | -7.6 |
| 263 | 121285761 | -7.6 |
| 264 | 49648154 | -7.6 |
| 265 | 26647725 | -7.6 |
| 266 | 24824581 | -7.6 |
| 267 | 4248393 | -7.6 |
| 268 | 24282846 | -7.6 |
| 269 | 24314215 | -7.6 |
| 270 | 4251176 | -7.6 |
| 271 | 56373850 | -7.6 |
| 272 | 124949344 | -7.6 |
| 273 | 87336130 | -7.6 |
| 274 | 24805576 | -7.6 |
| 275 | 22400822 | -7.6 |
| 276 | 17465823 | -7.6 |
| 277 | 17387661 | -7.6 |
| 278 | 26614769 | -7.6 |
| 279 | 24410578 | -7.6 |
| 280 | 24340347 | -7.6 |
| 281 | 26646865 | -7.6 |
| 282 | 57260942 | -7.6 |
| 283 | 26650477 | -7.6 |
| 284 | 24409780 | -7.6 |
| 285 | 17386483 | -7.6 |
| 286 | 49824701 | -7.6 |
| 287 | 24811051 | -7.6 |
| 288 | 57258695 | -7.6 |
| 289 | 847666 | -7.6 |
| 290 | 24352607 | -7.6 |
| 291 | 124360562 | -7.6 |
| 292 | 56315692 | -7.6 |
| 293 | 49719964 | -7.6 |
| 294 | 24831455 | -7.6 |
| 295 | 11536470 | -7.6 |
| 296 | 26534800 | -7.6 |
| 297 | 124949195 | -7.6 |
| 298 | 24834956 | -7.6 |
| 299 | 24343584 | -7.6 |
| 300 | 26696882 | -7.6 |
| 301 | 7967028 | -7.6 |
| 302 | 24779473 | -7.6 |
| 303 | 26618143 | -7.6 |
| 304 | 49730399 | -7.6 |
| 305 | 85199647 | -7.6 |
| 306 | 4244386 | -7.6 |
| 307 | 17514686 | -7.6 |
| 308 | 85271270 | -7.6 |
| 309 | 104222731 | -7.6 |
| 310 | 24388674 | -7.6 |
| 311 | 24373034 | -7.6 |
| 312 | 49829194 | -7.6 |
| 313 | 49672570 | -7.6 |
| 314 | 144095610 | -7.6 |
| 315 | 103075127 | -7.6 |
| 316 | 24339638 | -7.6 |
| 317 | 14745246 | -7.6 |
| 318 | 24378622 | -7.6 |
| 319 | 24287987 | -7.6 |
| 320 | 24826701 | -7.6 |
| 321 | 24805094 | -7.6 |
| 322 | 29216503 | -7.6 |
| 323 | 89854569 | -7.6 |
| 324 | 24403006 | -7.6 |
| 325 | 22413347 | -7.6 |
| 326 | 26533028 | -7.6 |
| 327 | 24839682 | -7.6 |
| 328 | 24311975 | -7.6 |
| 329 | 49647455 | -7.6 |
| 330 | 17412058 | -7.6 |
| 331 | 26620485 | -7.6 |
| 332 | 49736246 | -7.6 |
| 333 | 4256034 | -7.6 |
| 334 | 26648575 | -7.6 |
| 335 | 49642016 | -7.5 |
| 336 | 24406313 | -7.5 |
| 337 | 17409537 | -7.5 |
| 338 | 24807394 | -7.5 |
| 339 | 26731242 | -7.5 |
| 340 | 14740940 | -7.5 |
| 341 | 26636539 | -7.5 |
| 342 | 24794762 | -7.5 |
| 343 | 26540073 | -7.5 |
| 344 | 24296591 | -7.5 |
| 345 | 124949950 | -7.5 |
| 346 | 7964921 | -7.5 |
| 347 | 49672458 | -7.5 |
| 348 | 49736867 | -7.5 |
| 349 | 24303060 | -7.5 |
| 350 | 14745985 | -7.5 |
| 351 | 81066655 | -7.5 |
| 352 | 93375306 | -7.5 |
| 353 | 24337116 | -7.5 |
| 354 | 56316430 | -7.5 |
| 355 | 861467 | -7.5 |
| 356 | 24782980 | -7.5 |
| 357 | 57269190 | -7.5 |
| 358 | 17439005 | -7.5 |
| 359 | 49647425 | -7.5 |
| 360 | 26659530 | -7.5 |
| 361 | 26646589 | -7.5 |
| 362 | 14727433 | -7.5 |
| 363 | 26755333 | -7.5 |
| 364 | 85268439 | -7.5 |
| 365 | 24800652 | -7.5 |
| 366 | 56322790 | -7.5 |
| 367 | 26650979 | -7.5 |
| 368 | 51089566 | -7.5 |
| 369 | 26647682 | -7.5 |
| 370 | 26618003 | -7.5 |
| 371 | 57261346 | -7.5 |
| 372 | 24351964 | -7.5 |
| 373 | 49669804 | -7.5 |
| 374 | 47202582 | -7.5 |
| 375 | 24824509 | -7.5 |
| 376 | 24339240 | -7.5 |
| 377 | 49719215 | -7.5 |
| 378 | 47198965 | -7.5 |
| 379 | 49828068 | -7.5 |
| 380 | 24304203 | -7.5 |
| 381 | 24349056 | -7.5 |
| 382 | 49714246 | -7.5 |
| 383 | 14742290 | -7.5 |
| 384 | 26530026 | -7.5 |
| 385 | 24301698 | -7.5 |
| 386 | 24346826 | -7.5 |
| 387 | 49731385 | -7.5 |
| 388 | 56322410 | -7.5 |
| 389 | 57255548 | -7.5 |
| 390 | 26513963 | -7.5 |
| 391 | 162163293 | -7.5 |
| 392 | 24827282 | -7.5 |
| 393 | 24395345 | -7.5 |
| 394 | 24329802 | -7.5 |
| 395 | 14743346 | -7.5 |
| 396 | 24377599 | -7.5 |
| 397 | 49820660 | -7.5 |
| 398 | 24307983 | -7.5 |
| 399 | 24287689 | -7.5 |
| 400 | 49667112 | -7.5 |
| 401 | 24789674 | -7.5 |
| 402 | 4263584 | -7.5 |
| 403 | 24388875 | -7.5 |
| 404 | 26630120 | -7.5 |
| 405 | 56436750 | -7.5 |
| 406 | 49675442 | -7.5 |
| 407 | 125311436 | -7.5 |
| 408 | 121283341 | -7.5 |
| 409 | 103076027 | -7.5 |
| 410 | 7970748 | -7.5 |
| 411 | 24818842 | -7.5 |
| 412 | 26648015 | -7.5 |
| 413 | 74373975 | -7.5 |
| 414 | 24781396 | -7.5 |
| 415 | 74373988 | -7.5 |
| 416 | 57259556 | -7.5 |
| 417 | 26648668 | -7.5 |
| 418 | 4249062 | -7.5 |
| 419 | 26540165 | -7.5 |
| 420 | 49640654 | -7.5 |
| 421 | 24798475 | -7.5 |
| 422 | 26652673 | -7.5 |
| 423 | 87335739 | -7.5 |
| 424 | 56316263 | -7.5 |
| 425 | 3714019 | -7.5 |
| 426 | 85303730 | -7.5 |
| 427 | 57263885 | -7.5 |
| 428 | 848811 | -7.5 |
| 429 | 848763 | -7.5 |
| 430 | 851235 | -7.5 |
| 431 | 49720551 | -7.5 |
| 432 | 24336583 | -7.5 |
| 433 | 4258814 | -7.5 |
| 434 | 49827567 | -7.5 |
| 435 | 24282807 | -7.5 |
| 436 | 4243186 | -7.5 |
| 437 | 26650055 | -7.5 |
| 438 | 24837399 | -7.5 |
| 439 | 4257201 | -7.5 |
| 440 | 26617853 | -7.5 |
| 441 | 24782640 | -7.5 |
| 442 | 4247562 | -7.5 |
| 443 | 3717787 | -7.5 |
| 444 | 124350119 | -7.5 |
| 445 | 24333664 | -7.5 |
| 446 | 56314810 | -7.5 |
| 447 | 4255793 | -7.5 |
| 448 | 24416975 | -7.5 |
| 449 | 17440442 | -7.5 |
| 450 | 125305671 | -7.5 |
| 451 | 24384883 | -7.5 |
| 452 | 24782196 | -7.5 |
| 453 | 17478296 | -7.5 |
| 454 | 17512779 | -7.5 |
| 455 | 4249414 | -7.5 |
| 456 | 49737741 | -7.5 |
| 457 | 47197173 | -7.5 |
| 458 | 26617032 | -7.5 |
| 459 | 24794757 | -7.5 |
| 460 | 17452459 | -7.5 |
| 461 | 26736133 | -7.5 |
| 462 | 24404268 | -7.5 |
| 463 | 26756616 | -7.4 |
| 464 | 99454231 | -7.4 |
| 465 | 24829541 | -7.4 |
| 466 | 17449993 | -7.4 |
| 467 | 24390119 | -7.4 |
| 468 | 26538353 | -7.4 |
| 469 | 24301453 | -7.4 |
| 470 | 4242752 | -7.4 |
| 471 | 49819190 | -7.4 |
| 472 | 26650735 | -7.4 |
| 473 | 26646945 | -7.4 |
| 474 | 49818665 | -7.4 |
| 475 | 85146028 | -7.4 |
| 476 | 26646734 | -7.4 |
| 477 | 24306099 | -7.4 |
| 478 | 24387020 | -7.4 |
| 479 | 3712248 | -7.4 |
| 480 | 24269236 | -7.4 |
| 481 | 864607 | -7.4 |
| 482 | 24797941 | -7.4 |
| 483 | 24372913 | -7.4 |
| 484 | 24358364 | -7.4 |
| 485 | 49647605 | -7.4 |
| 486 | 99494461 | -7.4 |
| 487 | 26614060 | -7.4 |
| 488 | 50122263 | -7.4 |
| 489 | 85199183 | -7.4 |
| 490 | 49828235 | -7.4 |
| 491 | 864892 | -7.4 |
| 492 | 24834770 | -7.4 |
| 493 | 26648979 | -7.4 |
| 494 | 26648271 | -7.4 |
| 495 | 4251887 | -7.4 |
| 496 | 24779480 | -7.4 |
| 497 | 24306125 | -7.4 |
| 498 | 26535743 | -7.4 |
| 499 | 24320490 | -7.4 |
| 500 | 17474140 | -7.4 |
| 501 | 24342150 | -7.4 |
| 502 | 49722767 | -7.4 |
| 503 | 57264640 | -7.4 |
| 504 | 26529077 | -7.4 |
| 505 | 17484021 | -7.4 |
| 506 | 26650978 | -7.4 |
| 507 | 24328269 | -7.4 |
| 508 | 26659446 | -7.4 |
| 509 | 24332195 | -7.4 |
| 510 | 24366163 | -7.4 |
| 511 | 4243518 | -7.4 |
| 512 | 17483175 | -7.4 |
| 513 | 24782017 | -7.4 |
| 514 | 134465100 | -7.4 |
| 515 | 4246276 | -7.4 |
| 516 | 17516624 | -7.4 |
| 517 | 49642753 | -7.4 |
| 518 | 4256142 | -7.4 |
| 519 | 17468476 | -7.4 |
| 520 | 17407506 | -7.4 |
| 521 | 57264919 | -7.4 |
| 522 | 24800463 | -7.4 |
| 523 | 17468832 | -7.4 |
| 524 | 49674814 | -7.4 |
| 525 | 49646705 | -7.4 |
| 526 | 57267624 | -7.4 |
| 527 | 24395843 | -7.4 |
| 528 | 4257443 | -7.4 |
| 529 | 4258558 | -7.4 |
| 530 | 24327167 | -7.4 |
| 531 | 26533456 | -7.4 |
| 532 | 26731464 | -7.4 |
| 533 | 24350490 | -7.4 |
| 534 | 4251394 | -7.4 |
| 535 | 24813284 | -7.4 |
| 536 | 26613772 | -7.4 |
| 537 | 24372084 | -7.4 |
| 538 | 24291160 | -7.4 |
| 539 | 7968152 | -7.4 |
| 540 | 4262419 | -7.4 |
| 541 | 848308 | -7.4 |
| 542 | 14743222 | -7.4 |
| 543 | 24352016 | -7.4 |
| 544 | 24310485 | -7.4 |
| 545 | 861197 | -7.4 |
| 546 | 24322954 | -7.4 |
| 547 | 26626724 | -7.4 |
| 548 | 56316127 | -7.4 |
| 549 | 56319813 | -7.4 |
| 550 | 24397474 | -7.4 |
| 551 | 14747173 | -7.4 |
| 552 | 57259470 | -7.4 |
| 553 | 26614461 | -7.4 |
| 554 | 24344191 | -7.4 |
| 555 | 17387060 | -7.4 |
| 556 | 103059875 | -7.4 |
| 557 | 24823017 | -7.4 |
| 558 | 865490 | -7.4 |
| 559 | 17459610 | -7.4 |
| 560 | 49727956 | -7.4 |
| 561 | 85269995 | -7.4 |
| 562 | 17465028 | -7.4 |
| 563 | 26615242 | -7.4 |
| 564 | 87344727 | -7.4 |
| 565 | 26538220 | -7.4 |
| 566 | 24415713 | -7.4 |
| 567 | 74373759 | -7.4 |
| 568 | 4255939 | -7.4 |
| 569 | 24818902 | -7.4 |
| 570 | 26632911 | -7.4 |
| 571 | 144096840 | -7.4 |
| 572 | 26513823 | -7.4 |
| 573 | 24409825 | -7.4 |
| 574 | 144223378 | -7.4 |
| 575 | 24821068 | -7.4 |
| 576 | 50100911 | -7.4 |
| 577 | 24796608 | -7.4 |
| 578 | 124896714 | -7.4 |
| 579 | 24799224 | -7.4 |
| 580 | 56318192 | -7.4 |
| 581 | 24272815 | -7.4 |
| 582 | 49732401 | -7.4 |
| 583 | 3712633 | -7.4 |
| 584 | 24797988 | -7.4 |
| 585 | 26537956 | -7.4 |
| 586 | 3712326 | -7.4 |
| 587 | 49827551 | -7.4 |
| 588 | 26730744 | -7.4 |
| 589 | 26541061 | -7.4 |
| 590 | 124755588 | -7.4 |
| 591 | 24797784 | -7.4 |
| 592 | 24824874 | -7.4 |
| 593 | 26536798 | -7.4 |
| 594 | 124948956 | -7.4 |
| 595 | 14735625 | -7.4 |
| 596 | 3714170 | -7.4 |
| 597 | 49718279 | -7.4 |
| 598 | 17485628 | -7.4 |
| 599 | 124949879 | -7.4 |
| 600 | 24318455 | -7.4 |
| 601 | 14738791 | -7.4 |
| 602 | 22412818 | -7.4 |
| 603 | 26617040 | -7.4 |
| 604 | 26615745 | -7.4 |
| 605 | 26730541 | -7.4 |
| 606 | 24405047 | -7.4 |
| 607 | 26614675 | -7.4 |
| 608 | 3716846 | -7.4 |
| 609 | 24785038 | -7.4 |
| 610 | 85176593 | -7.4 |
| 611 | 24799322 | -7.4 |
| 612 | 14721630 | -7.3 |
| 613 | 24371438 | -7.3 |
| 614 | 16953518 | -7.3 |
| 615 | 17415901 | -7.3 |
| 616 | 7973360 | -7.3 |
| 617 | 7964606 | -7.3 |
| 618 | 26651180 | -7.3 |
| 619 | 24283655 | -7.3 |
| 620 | 22414222 | -7.3 |
| 621 | 26740871 | -7.3 |
| 622 | 24801351 | -7.3 |
| 623 | 14746474 | -7.3 |
| 624 | 26662555 | -7.3 |
| 625 | 26636245 | -7.3 |
| 626 | 24789184 | -7.3 |
| 627 | 24800685 | -7.3 |
| 628 | 85145949 | -7.3 |
| 629 | 24275890 | -7.3 |
| 630 | 26535348 | -7.3 |
| 631 | 85148315 | -7.3 |
| 632 | 26650232 | -7.3 |
| 633 | 99356498 | -7.3 |
| 634 | 56316009 | -7.3 |
| 635 | 26746807 | -7.3 |
| 636 | 49645908 | -7.3 |
| 637 | 26649160 | -7.3 |
| 638 | 24781585 | -7.3 |
| 639 | 57268731 | -7.3 |
| 640 | 49679989 | -7.3 |
| 641 | 26539595 | -7.3 |
| 642 | 26724398 | -7.3 |
| 643 | 49667215 | -7.3 |
| 644 | 24837915 | -7.3 |
| 645 | 51085377 | -7.3 |
| 646 | 24352301 | -7.3 |
| 647 | 24820007 | -7.3 |
| 648 | 26542566 | -7.3 |
| 649 | 24824723 | -7.3 |
| 650 | 26542024 | -7.3 |
| 651 | 85199391 | -7.3 |
| 652 | 3715126 | -7.3 |
| 653 | 99360519 | -7.3 |
| 654 | 17475678 | -7.3 |
| 655 | 14739406 | -7.3 |
| 656 | 7965657 | -7.3 |
| 657 | 22405342 | -7.3 |
| 658 | 144097183 | -7.3 |
| 659 | 49717845 | -7.3 |
| 660 | 24404959 | -7.3 |
| 661 | 144095651 | -7.3 |
| 662 | 24832610 | -7.3 |
| 663 | 49678643 | -7.3 |
| 664 | 85176812 | -7.3 |
| 665 | 14738911 | -7.3 |
| 666 | 49826402 | -7.3 |
| 667 | 56319490 | -7.3 |
| 668 | 57262645 | -7.3 |
| 669 | 17409540 | -7.3 |
| 670 | 26618614 | -7.3 |
| 671 | 24411853 | -7.3 |
| 672 | 26651276 | -7.3 |
| 673 | 104224575 | -7.3 |
| 674 | 17513366 | -7.3 |
| 675 | 51089220 | -7.3 |
| 676 | 14745236 | -7.3 |
| 677 | 17515341 | -7.3 |
| 678 | 57265246 | -7.3 |
| 679 | 47196830 | -7.3 |
| 680 | 17514576 | -7.3 |
| 681 | 22406228 | -7.3 |
| 682 | 24802282 | -7.3 |
| 683 | 47199745 | -7.3 |
| 684 | 26730308 | -7.3 |
| 685 | 26617784 | -7.3 |
| 686 | 99455298 | -7.3 |
| 687 | 17387572 | -7.3 |
| 688 | 49722457 | -7.3 |
| 689 | 14731248 | -7.3 |
| 690 | 26531587 | -7.3 |
| 691 | 24316631 | -7.3 |
| 692 | 26613573 | -7.3 |
| 693 | 144091349 | -7.3 |
| 694 | 49723208 | -7.3 |
| 695 | 49645356 | -7.3 |
| 696 | 26613560 | -7.3 |
| 697 | 49670175 | -7.3 |
| 698 | 24814875 | -7.3 |
| 699 | 24345849 | -7.3 |
| 700 | 50110359 | -7.3 |
| 701 | 4256928 | -7.3 |
| 702 | 26671103 | -7.3 |
| 703 | 124949758 | -7.3 |
| 704 | 56321901 | -7.3 |
| 705 | 4256132 | -7.3 |
| 706 | 24783630 | -7.3 |
| 707 | 3715251 | -7.3 |
| 708 | 844780 | -7.3 |
| 709 | 24392716 | -7.3 |
| 710 | 24816897 | -7.3 |
| 711 | 85148803 | -7.3 |
| 712 | 14721462 | -7.3 |
| 713 | 26540287 | -7.3 |
| 714 | 24833804 | -7.3 |
| 715 | 121286204 | -7.3 |
| 716 | 17438727 | -7.3 |
| 717 | 49721606 | -7.3 |
| 718 | 26731628 | -7.3 |
| 719 | 49642990 | -7.3 |
| 720 | 4243007 | -7.3 |
| 721 | 99358203 | -7.3 |
| 722 | 17478048 | -7.3 |
| 723 | 14745075 | -7.3 |
| 724 | 24341373 | -7.3 |
| 725 | 17410725 | -7.3 |
| 726 | 24350566 | -7.3 |
| 727 | 4261452 | -7.3 |
| 728 | 17443839 | -7.3 |
| 729 | 26530424 | -7.3 |
| 730 | 4250958 | -7.3 |
| 731 | 17506126 | -7.3 |
| 732 | 26661767 | -7.3 |
| 733 | 121285573 | -7.3 |
| 734 | 24298282 | -7.3 |
| 735 | 24310121 | -7.3 |
| 736 | 26729903 | -7.3 |
| 737 | 17509945 | -7.3 |
| 738 | 124349909 | -7.3 |
| 739 | 17507450 | -7.3 |
| 740 | 49719655 | -7.3 |
| 741 | 11536483 | -7.3 |
| 742 | 865112 | -7.3 |
| 743 | 24824145 | -7.3 |
| 744 | 49828871 | -7.3 |
| 745 | 24832836 | -7.3 |
| 746 | 24792592 | -7.3 |
| 747 | 49826761 | -7.3 |
| 748 | 26727266 | -7.3 |
| 749 | 49645361 | -7.3 |
| 750 | 124947953 | -7.3 |
| 751 | 124948833 | -7.3 |
| 752 | 49641312 | -7.3 |
| 753 | 853379 | -7.3 |
| 754 | 3713611 | -7.3 |
| 755 | 26649539 | -7.3 |
| 756 | 124350134 | -7.3 |
| 757 | 24808391 | -7.3 |
| 758 | 96021301 | -7.3 |
| 759 | 49821532 | -7.3 |
| 760 | 26648601 | -7.3 |
| 761 | 3716438 | -7.3 |
| 762 | 26650688 | -7.3 |
| 763 | 124949939 | -7.3 |
| 764 | 24354982 | -7.3 |
| 765 | 89855298 | -7.3 |
| 766 | 49816195 | -7.3 |
| 767 | 24363301 | -7.3 |
| 768 | 57262151 | -7.3 |
| 769 | 24802135 | -7.3 |
| 770 | 24800317 | -7.3 |
| 771 | 24416605 | -7.3 |
| 772 | 26620360 | -7.3 |
| 773 | 57263196 | -7.3 |
| 774 | 26659929 | -7.3 |
| 775 | 24833268 | -7.3 |
| 776 | 124949219 | -7.3 |
| 777 | 49676071 | -7.3 |
| 778 | 24832459 | -7.3 |
| 779 | 24283307 | -7.3 |
| 780 | 49665925 | -7.3 |
| 781 | 49736308 | -7.3 |
| 782 | 24799956 | -7.3 |
| 783 | 24832207 | -7.3 |
| 784 | 22410720 | -7.3 |
| 785 | 46500047 | -7.3 |
| 786 | 842396 | -7.3 |
| 787 | 26633731 | -7.3 |
| 788 | 24289973 | -7.3 |
| 789 | 24821089 | -7.3 |
| 790 | 49734116 | -7.3 |
| 791 | 17509286 | -7.3 |
| 792 | 24336476 | -7.3 |
| 793 | 17447965 | -7.3 |
| 794 | 17434019 | -7.3 |
| 795 | 24389741 | -7.3 |
| 796 | 3711913 | -7.3 |
| 797 | 26536077 | -7.3 |
| 798 | 24293650 | -7.3 |
| 799 | 26664443 | -7.3 |
| 800 | 49734834 | -7.3 |
| 801 | 99495232 | -7.3 |
| 802 | 49680384 | -7.3 |
| 803 | 99357755 | -7.3 |
| 804 | 49723222 | -7.3 |
| 805 | 56373488 | -7.3 |
| 806 | 26649722 | -7.3 |
| 807 | 24831842 | -7.3 |
| 808 | 24808993 | -7.3 |
| 809 | 26619875 | -7.3 |
| 810 | 81066580 | -7.3 |
| 811 | 26622017 | -7.3 |
| 812 | 7973082 | -7.3 |
| 813 | 56318584 | -7.3 |
| 814 | 24798810 | -7.3 |
| 815 | 7968009 | -7.3 |
| 816 | 144098062 | -7.3 |
| 817 | 17516857 | -7.3 |
| 818 | 104224583 | -7.3 |
| 819 | 24319558 | -7.3 |
| 820 | 24286125 | -7.3 |
| 821 | 144089289 | -7.3 |
| 822 | 56316690 | -7.3 |
| 823 | 85149123 | -7.2 |
| 824 | 24824526 | -7.2 |
| 825 | 26543104 | -7.2 |
| 826 | 17455973 | -7.2 |
| 827 | 14736413 | -7.2 |
| 828 | 49668038 | -7.2 |
| 829 | 17508604 | -7.2 |
| 830 | 24827023 | -7.2 |
| 831 | 121283528 | -7.2 |
| 832 | 24782689 | -7.2 |
| 833 | 24335068 | -7.2 |
| 834 | 24300467 | -7.2 |
| 835 | 24832130 | -7.2 |
| 836 | 24401327 | -7.2 |
| 837 | 24335249 | -7.2 |
| 838 | 4246655 | -7.2 |
| 839 | 22410181 | -7.2 |
| 840 | 7968853 | -7.2 |
| 841 | 26670650 | -7.2 |
| 842 | 24351373 | -7.2 |
| 843 | 49676292 | -7.2 |
| 844 | 99495546 | -7.2 |
| 845 | 3716193 | -7.2 |
| 846 | 26615839 | -7.2 |
| 847 | 24837261 | -7.2 |
| 848 | 49679961 | -7.2 |
| 849 | 24820171 | -7.2 |
| 850 | 17431592 | -7.2 |
| 851 | 24824580 | -7.2 |
| 852 | 4245765 | -7.2 |
| 853 | 26666730 | -7.2 |
| 854 | 865412 | -7.2 |
| 855 | 26647515 | -7.2 |
| 856 | 51085899 | -7.2 |
| 857 | 24787915 | -7.2 |
| 858 | 24832223 | -7.2 |
| 859 | 4251156 | -7.2 |
| 860 | 24335975 | -7.2 |
| 861 | 26746803 | -7.2 |
| 862 | 22404062 | -7.2 |
| 863 | 846409 | -7.2 |
| 864 | 24322874 | -7.2 |
| 865 | 29217513 | -7.2 |
| 866 | 856034 | -7.2 |
| 867 | 856323 | -7.2 |
| 868 | 4261818 | -7.2 |
| 869 | 49645140 | -7.2 |
| 870 | 24359404 | -7.2 |
| 871 | 24429178 | -7.2 |
| 872 | 26535687 | -7.2 |
| 873 | 24803950 | -7.2 |
| 874 | 104233114 | -7.2 |
| 875 | 3715481 | -7.2 |
| 876 | 124756780 | -7.2 |
| 877 | 47201037 | -7.2 |
| 878 | 26531025 | -7.2 |
| 879 | 87346004 | -7.2 |
| 880 | 24284845 | -7.2 |
| 881 | 14733104 | -7.2 |
| 882 | 99495482 | -7.2 |
| 883 | 49676817 | -7.2 |
| 884 | 49730620 | -7.2 |
| 885 | 26648420 | -7.2 |
| 886 | 85269935 | -7.2 |
| 887 | 49816455 | -7.2 |
| 888 | 24789230 | -7.2 |
| 889 | 57260646 | -7.2 |
| 890 | 14739007 | -7.2 |
| 891 | 17476743 | -7.2 |
| 892 | 24328815 | -7.2 |
| 893 | 7967650 | -7.2 |
| 894 | 24787154 | -7.2 |
| 895 | 99494828 | -7.2 |
| 896 | 49680219 | -7.2 |
| 897 | 24408750 | -7.2 |
| 898 | 4251939 | -7.2 |
| 899 | 26641116 | -7.2 |
| 900 | 14745839 | -7.2 |
| 901 | 24791828 | -7.2 |
| 902 | 26730752 | -7.2 |
| 903 | 24348580 | -7.2 |
| 904 | 24411805 | -7.2 |
| 905 | 24836987 | -7.2 |
| 906 | 49671263 | -7.2 |
| 907 | 24284072 | -7.2 |
| 908 | 26662001 | -7.2 |
| 909 | 57261168 | -7.2 |
| 910 | 144096796 | -7.2 |
| 911 | 24810837 | -7.2 |
| 912 | 24794977 | -7.2 |
| 913 | 144097666 | -7.2 |
| 914 | 4247282 | -7.2 |
| 915 | 4263187 | -7.2 |
| 916 | 3712967 | -7.2 |
| 917 | 81066331 | -7.2 |
| 918 | 24413126 | -7.2 |
| 919 | 124756712 | -7.2 |
| 920 | 852985 | -7.2 |
| 921 | 24393480 | -7.2 |
| 922 | 136889460 | -7.2 |
| 923 | 26730430 | -7.2 |
| 924 | 22408109 | -7.2 |
| 925 | 16953805 | -7.2 |
| 926 | 26620883 | -7.2 |
| 927 | 14726206 | -7.2 |
| 928 | 3713313 | -7.2 |
| 929 | 865595 | -7.2 |
| 930 | 847663 | -7.2 |
| 931 | 24385383 | -7.2 |
| 932 | 57263248 | -7.2 |
| 933 | 24385377 | -7.2 |
| 934 | 104233329 | -7.2 |
| 935 | 24794485 | -7.2 |
| 936 | 4254738 | -7.2 |
| 937 | 26528676 | -7.2 |
| 938 | 7976683 | -7.2 |
| 939 | 26669756 | -7.2 |
| 940 | 24374502 | -7.2 |
| 941 | 24406309 | -7.2 |
| 942 | 49671749 | -7.2 |
| 943 | 24794900 | -7.2 |
| 944 | 24288509 | -7.2 |
| 945 | 49821201 | -7.2 |
| 946 | 26640087 | -7.2 |
| 947 | 24841501 | -7.2 |
| 948 | 57263359 | -7.2 |
| 949 | 24365366 | -7.2 |
| 950 | 49641641 | -7.2 |
| 951 | 7997907 | -7.2 |
| 952 | 24279139 | -7.2 |
| 953 | 24819104 | -7.2 |
| 954 | 26614125 | -7.2 |
| 955 | 26628465 | -7.2 |
| 956 | 26649911 | -7.2 |
| 957 | 853664 | -7.2 |
| 958 | 17466798 | -7.2 |
| 959 | 22413784 | -7.2 |
| 960 | 24322902 | -7.2 |
| 961 | 24342332 | -7.2 |
| 962 | 26648948 | -7.2 |
| 963 | 47201072 | -7.2 |
| 964 | 24313956 | -7.2 |
| 965 | 26726041 | -7.2 |
| 966 | 57265811 | -7.2 |
| 967 | 24785934 | -7.2 |
| 968 | 49646776 | -7.2 |
| 969 | 24282388 | -7.2 |
| 970 | 152199731 | -7.2 |
| 971 | 24387451 | -7.2 |
| 972 | 104224747 | -7.2 |
| 973 | 7967725 | -7.2 |
| 974 | 49719059 | -7.2 |
| 975 | 89855804 | -7.2 |
| 976 | 57255974 | -7.2 |
| 977 | 57262332 | -7.2 |
| 978 | 49718093 | -7.2 |
| 979 | 862385 | -7.2 |
| 980 | 4242166 | -7.2 |
| 981 | 4255086 | -7.2 |
| 982 | 89851222 | -7.2 |
| 983 | 24343866 | -7.2 |
| 984 | 24800500 | -7.2 |
| 985 | 842527 | -7.2 |
| 986 | 85148804 | -7.2 |
| 987 | 24812626 | -7.2 |
| 988 | 3715356 | -7.2 |
| 989 | 22409075 | -7.2 |
| 990 | 14728756 | -7.2 |
| 991 | 17385841 | -7.2 |
| 992 | 22415916 | -7.2 |
| 993 | 26752630 | -7.2 |
| 994 | 26535797 | -7.2 |
| 995 | 4262223 | -7.2 |
| 996 | 26649754 | -7.2 |
| 997 | 24412540 | -7.2 |
| 998 | 99358243 | -7.2 |
| 999 | 49667357 | -7.2 |
| 1000 | 26537703 | -7.2 |
| 1001 | 14741574 | -7.2 |
| 1002 | 7965224 | -7.2 |
| 1003 | 124349835 | -7.2 |
| 1004 | 26535646 | -7.2 |
| 1005 | 4245280 | -7.2 |
| 1006 | 14735923 | -7.2 |
| 1007 | 26660348 | -7.2 |
| 1008 | 125306859 | -7.2 |
| 1009 | 24317940 | -7.2 |
| 1010 | 26729305 | -7.2 |
| 1011 | 24831993 | -7.2 |
| 1012 | 87550771 | -7.2 |
| 1013 | 26651058 | -7.2 |
| 1014 | 85273871 | -7.2 |
| 1015 | 26624366 | -7.2 |
| 1016 | 24408092 | -7.2 |
| 1017 | 49825354 | -7.2 |
| 1018 | 26752369 | -7.2 |
| 1019 | 24406803 | -7.2 |
| 1020 | 24807303 | -7.2 |
| 1021 | 51090681 | -7.2 |
| 1022 | 99356266 | -7.2 |
| 1023 | 24825980 | -7.2 |
| 1024 | 103060642 | -7.2 |
| 1025 | 24808465 | -7.2 |
| 1026 | 134216045 | -7.2 |
| 1027 | 49676418 | -7.2 |
| 1028 | 850419 | -7.2 |
| 1029 | 49674491 | -7.2 |
| 1030 | 24825796 | -7.2 |
| 1031 | 49736871 | -7.2 |
| 1032 | 17452456 | -7.2 |
| 1033 | 864188 | -7.2 |
| 1034 | 24799942 | -7.2 |
| 1035 | 3716264 | -7.2 |
| 1036 | 49672664 | -7.2 |
| 1037 | 7967999 | -7.2 |
| 1038 | 26662791 | -7.2 |
| 1039 | 24783609 | -7.2 |
| 1040 | 103050353 | -7.2 |
| 1041 | 47202608 | -7.2 |
| 1042 | 14736138 | -7.2 |
| 1043 | 17455170 | -7.1 |
| 1044 | 49649004 | -7.1 |
| 1045 | 14734737 | -7.1 |
| 1046 | 24396956 | -7.1 |
| 1047 | 11535328 | -7.1 |
| 1048 | 85198494 | -7.1 |
| 1049 | 26542817 | -7.1 |
| 1050 | 49675687 | -7.1 |
| 1051 | 26648530 | -7.1 |
| 1052 | 26648881 | -7.1 |
| 1053 | 22415598 | -7.1 |
| 1054 | 49647503 | -7.1 |
| 1055 | 24335595 | -7.1 |
| 1056 | 3713299 | -7.1 |
| 1057 | 26634305 | -7.1 |
| 1058 | 24354071 | -7.1 |
| 1059 | 24368838 | -7.1 |
| 1060 | 24823573 | -7.1 |
| 1061 | 24831957 | -7.1 |
| 1062 | 11535235 | -7.1 |
| 1063 | 26624355 | -7.1 |
| 1064 | 26620053 | -7.1 |
| 1065 | 14731936 | -7.1 |
| 1066 | 3717640 | -7.1 |
| 1067 | 4246155 | -7.1 |
| 1068 | 17467682 | -7.1 |
| 1069 | 26537504 | -7.1 |
| 1070 | 26543224 | -7.1 |
| 1071 | 104224688 | -7.1 |
| 1072 | 49721215 | -7.1 |
| 1073 | 26528786 | -7.1 |
| 1074 | 26536999 | -7.1 |
| 1075 | 24818381 | -7.1 |
| 1076 | 24406753 | -7.1 |
| 1077 | 24285015 | -7.1 |
| 1078 | 851647 | -7.1 |
| 1079 | 26662202 | -7.1 |
| 1080 | 26662838 | -7.1 |
| 1081 | 24299604 | -7.1 |
| 1082 | 24825727 | -7.1 |
| 1083 | 49667154 | -7.1 |
| 1084 | 24337336 | -7.1 |
| 1085 | 14727381 | -7.1 |
| 1086 | 49724481 | -7.1 |
| 1087 | 49714316 | -7.1 |
| 1088 | 103075251 | -7.1 |
| 1089 | 56315809 | -7.1 |
| 1090 | 51086702 | -7.1 |
| 1091 | 51085905 | -7.1 |
| 1092 | 22415722 | -7.1 |
| 1093 | 56322971 | -7.1 |
| 1094 | 3717573 | -7.1 |
| 1095 | 99456999 | -7.1 |
| 1096 | 3713016 | -7.1 |
| 1097 | 11532923 | -7.1 |
| 1098 | 56323234 | -7.1 |
| 1099 | 4242894 | -7.1 |
| 1100 | 85147432 | -7.1 |
| 1101 | 57259075 | -7.1 |
| 1102 | 24395342 | -7.1 |
| 1103 | 26618354 | -7.1 |
| 1104 | 49829182 | -7.1 |
| 1105 | 49647392 | -7.1 |
| 1106 | 103051054 | -7.1 |
| 1107 | 17474323 | -7.1 |
| 1108 | 124349929 | -7.1 |
| 1109 | 24368951 | -7.1 |
| 1110 | 135383452 | -7.1 |
| 1111 | 26641771 | -7.1 |
| 1112 | 26646938 | -7.1 |
| 1113 | 26617932 | -7.1 |
| 1114 | 851277 | -7.1 |
| 1115 | 4245822 | -7.1 |
| 1116 | 26729838 | -7.1 |
| 1117 | 17455954 | -7.1 |
| 1118 | 22408716 | -7.1 |
| 1119 | 24290673 | -7.1 |
| 1120 | 24831986 | -7.1 |
| 1121 | 24296652 | -7.1 |
| 1122 | 26647082 | -7.1 |
| 1123 | 24836659 | -7.1 |
| 1124 | 14743580 | -7.1 |
| 1125 | 56318860 | -7.1 |
| 1126 | 47196822 | -7.1 |
| 1127 | 103060502 | -7.1 |
| 1128 | 857347 | -7.1 |
| 1129 | 26730352 | -7.1 |
| 1130 | 85198802 | -7.1 |
| 1131 | 24783790 | -7.1 |
| 1132 | 24323033 | -7.1 |
| 1133 | 103158879 | -7.1 |
| 1134 | 26624876 | -7.1 |
| 1135 | 49732150 | -7.1 |
| 1136 | 22401624 | -7.1 |
| 1137 | 24783806 | -7.1 |
| 1138 | 24350119 | -7.1 |
| 1139 | 57265417 | -7.1 |
| 1140 | 26651425 | -7.1 |
| 1141 | 24339467 | -7.1 |
| 1142 | 14725524 | -7.1 |
| 1143 | 24832714 | -7.1 |
| 1144 | 24266964 | -7.1 |
| 1145 | 26615066 | -7.1 |
| 1146 | 24812414 | -7.1 |
| 1147 | 26529129 | -7.1 |
| 1148 | 24292183 | -7.1 |
| 1149 | 51085581 | -7.1 |
| 1150 | 4247799 | -7.1 |
| 1151 | 24833202 | -7.1 |
| 1152 | 57263271 | -7.1 |
| 1153 | 124753620 | -7.1 |
| 1154 | 124951241 | -7.1 |
| 1155 | 17470352 | -7.1 |
| 1156 | 26730322 | -7.1 |
| 1157 | 26646639 | -7.1 |
| 1158 | 24369492 | -7.1 |
| 1159 | 49819149 | -7.1 |
| 1160 | 859008 | -7.1 |
| 1161 | 3717068 | -7.1 |
| 1162 | 24808367 | -7.1 |
| 1163 | 11114010 | -7.1 |
| 1164 | 16953875 | -7.1 |
| 1165 | 24802158 | -7.1 |
| 1166 | 7997755 | -7.1 |
| 1167 | 92763817 | -7.1 |
| 1168 | 24805699 | -7.1 |
| 1169 | 4243944 | -7.1 |
| 1170 | 24281851 | -7.1 |
| 1171 | 17510312 | -7.1 |
| 1172 | 85270439 | -7.1 |
| 1173 | 17402331 | -7.1 |
| 1174 | 56319432 | -7.1 |
| 1175 | 3714549 | -7.1 |
| 1176 | 103060518 | -7.1 |
| 1177 | 24820546 | -7.1 |
| 1178 | 85270411 | -7.1 |
| 1179 | 24307216 | -7.1 |
| 1180 | 14724283 | -7.1 |
| 1181 | 3716217 | -7.1 |
| 1182 | 24817650 | -7.1 |
| 1183 | 26614842 | -7.1 |
| 1184 | 24781266 | -7.1 |
| 1185 | 49823243 | -7.1 |
| 1186 | 92764332 | -7.1 |
| 1187 | 57263612 | -7.1 |
| 1188 | 850616 | -7.1 |
| 1189 | 49680812 | -7.1 |
| 1190 | 49676833 | -7.1 |
| 1191 | 47203611 | -7.1 |
| 1192 | 24396957 | -7.1 |
| 1193 | 26729159 | -7.1 |
| 1194 | 24282465 | -7.1 |
| 1195 | 49649752 | -7.1 |
| 1196 | 24408066 | -7.1 |
| 1197 | 7967760 | -7.1 |
| 1198 | 26617775 | -7.1 |
| 1199 | 47200099 | -7.1 |
| 1200 | 17468647 | -7.1 |
| 1201 | 24327576 | -7.1 |
| 1202 | 861164 | -7.1 |
| 1203 | 26620976 | -7.1 |
| 1204 | 24339418 | -7.1 |
| 1205 | 57266633 | -7.1 |
| 1206 | 29217543 | -7.1 |
| 1207 | 14726733 | -7.1 |
| 1208 | 26637370 | -7.1 |
| 1209 | 49817072 | -7.1 |
| 1210 | 26536312 | -7.1 |
| 1211 | 14729452 | -7.1 |
| 1212 | 14746751 | -7.1 |
| 1213 | 7965998 | -7.1 |
| 1214 | 26651290 | -7.1 |
| 1215 | 22407643 | -7.1 |
| 1216 | 144096195 | -7.1 |
| 1217 | 3713304 | -7.1 |
| 1218 | 49679884 | -7.1 |
| 1219 | 24395622 | -7.1 |
| 1220 | 24826416 | -7.1 |
| 1221 | 26614218 | -7.1 |
| 1222 | 26647510 | -7.1 |
| 1223 | 56319068 | -7.1 |
| 1224 | 24787457 | -7.1 |
| 1225 | 57263386 | -7.1 |
| 1226 | 4247177 | -7.1 |
| 1227 | 3716673 | -7.1 |
| 1228 | 26542724 | -7.1 |
| 1229 | 49649248 | -7.1 |
| 1230 | 3717158 | -7.1 |
| 1231 | 26731227 | -7.1 |
| 1232 | 24826757 | -7.1 |
| 1233 | 26542731 | -7.1 |
| 1234 | 17470481 | -7.1 |
| 1235 | 17485719 | -7.1 |
| 1236 | 24403926 | -7.1 |
| 1237 | 24410339 | -7.1 |
| 1238 | 49672427 | -7.1 |
| 1239 | 81066065 | -7.1 |
| 1240 | 134216038 | -7.1 |
| 1241 | 24783876 | -7.1 |
| 1242 | 844695 | -7.1 |
| 1243 | 4248190 | -7.1 |
| 1244 | 22401497 | -7.1 |
| 1245 | 99495661 | -7.1 |
| 1246 | 26531641 | -7.1 |
| 1247 | 56323072 | -7.1 |
| 1248 | 22416160 | -7.1 |
| 1249 | 103075975 | -7.1 |
| 1250 | 57267046 | -7.1 |
| 1251 | 49678789 | -7.1 |
| 1252 | 24362684 | -7.1 |
| 1253 | 3712705 | -7.1 |
| 1254 | 47198704 | -7.1 |
| 1255 | 24371067 | -7.1 |
| 1256 | 26639102 | -7.1 |
| 1257 | 99356390 | -7.1 |
| 1258 | 17409028 | -7.1 |
| 1259 | 26617995 | -7.1 |
| 1260 | 17446753 | -7.1 |
| 1261 | 49722974 | -7.1 |
| 1262 | 847789 | -7.1 |
| 1263 | 49721931 | -7.1 |
| 1264 | 4260626 | -7.1 |
| 1265 | 24799055 | -7.1 |
| 1266 | 24301246 | -7.1 |
| 1267 | 24412414 | -7.1 |
| 1268 | 49670168 | -7.1 |
| 1269 | 134216231 | -7.1 |
| 1270 | 14731028 | -7.1 |
| 1271 | 89853743 | -7.1 |
| 1272 | 47198528 | -7.1 |
| 1273 | 7966816 | -7.1 |
| 1274 | 26657471 | -7.1 |
| 1275 | 14743795 | -7.1 |
| 1276 | 24320522 | -7.1 |
| 1277 | 49672150 | -7.1 |
| 1278 | 14744640 | -7.1 |
| 1279 | 17464196 | -7.1 |
| 1280 | 47199794 | -7.1 |
| 1281 | 17387811 | -7.1 |
| 1282 | 24347618 | -7.1 |
| 1283 | 24405548 | -7.1 |
| 1284 | 14732641 | -7.1 |
| 1285 | 4252361 | -7.1 |
| 1286 | 57258554 | -7.1 |
| 1287 | 26611865 | -7.1 |
| 1288 | 24308819 | -7.1 |
| 1289 | 17401913 | -7.1 |
| 1290 | 24792638 | -7.1 |
| 1291 | 49731618 | -7.1 |
| 1292 | 26542313 | -7.1 |
| 1293 | 14723694 | -7.1 |
| 1294 | 49823425 | -7.1 |
| 1295 | 26728909 | -7.1 |
| 1296 | 862896 | -7.1 |
| 1297 | 24390783 | -7.1 |
| 1298 | 24289472 | -7.1 |
| 1299 | 57258161 | -7.1 |
| 1300 | 16953984 | -7.1 |
| 1301 | 17411435 | -7.1 |
| 1302 | 24405291 | -7.1 |
| 1303 | 857448 | -7.1 |
| 1304 | 3713860 | -7.1 |
| 1305 | 17402002 | -7.1 |
| 1306 | 24823982 | -7.1 |
| 1307 | 26538820 | -7.1 |
| 1308 | 85270757 | -7 |
| 1309 | 3715360 | -7 |
| 1310 | 50112891 | -7 |
| 1311 | 24785999 | -7 |
| 1312 | 85271716 | -7 |
| 1313 | 22416612 | -7 |
| 1314 | 24831707 | -7 |
| 1315 | 49649545 | -7 |
| 1316 | 26646564 | -7 |
| 1317 | 49826575 | -7 |
| 1318 | 85199738 | -7 |
| 1319 | 26645547 | -7 |
| 1320 | 24817019 | -7 |
| 1321 | 24341405 | -7 |
| 1322 | 24830027 | -7 |
| 1323 | 26725730 | -7 |
| 1324 | 47201149 | -7 |
| 1325 | 14746793 | -7 |
| 1326 | 26730388 | -7 |
| 1327 | 24394262 | -7 |
| 1328 | 49676065 | -7 |
| 1329 | 26625534 | -7 |
| 1330 | 17479712 | -7 |
| 1331 | 14735657 | -7 |
| 1332 | 124880149 | -7 |
| 1333 | 89850468 | -7 |
| 1334 | 103050220 | -7 |
| 1335 | 24794227 | -7 |
| 1336 | 852001 | -7 |
| 1337 | 49674508 | -7 |
| 1338 | 26757640 | -7 |
| 1339 | 26539197 | -7 |
| 1340 | 126496941 | -7 |
| 1341 | 17449021 | -7 |
| 1342 | 26647782 | -7 |
| 1343 | 56405569 | -7 |
| 1344 | 17411660 | -7 |
| 1345 | 16952787 | -7 |
| 1346 | 24385666 | -7 |
| 1347 | 49732503 | -7 |
| 1348 | 4260639 | -7 |
| 1349 | 26651434 | -7 |
| 1350 | 26659011 | -7 |
| 1351 | 26532221 | -7 |
| 1352 | 14718952 | -7 |
| 1353 | 103162652 | -7 |
| 1354 | 7999853 | -7 |
| 1355 | 17444138 | -7 |
| 1356 | 49675259 | -7 |
| 1357 | 26537220 | -7 |
| 1358 | 26614165 | -7 |
| 1359 | 26541296 | -7 |
| 1360 | 50086334 | -7 |
| 1361 | 24837738 | -7 |
| 1362 | 99360740 | -7 |
| 1363 | 99357106 | -7 |
| 1364 | 24428670 | -7 |
| 1365 | 862504 | -7 |
| 1366 | 85200269 | -7 |
| 1367 | 49641319 | -7 |
| 1368 | 24812287 | -7 |
| 1369 | 57255629 | -7 |
| 1370 | 49722844 | -7 |
| 1371 | 24334311 | -7 |
| 1372 | 49669849 | -7 |
| 1373 | 24796602 | -7 |
| 1374 | 17413400 | -7 |
| 1375 | 4242499 | -7 |
| 1376 | 103158989 | -7 |
| 1377 | 57260008 | -7 |
| 1378 | 85198151 | -7 |
| 1379 | 26730303 | -7 |
| 1380 | 49671197 | -7 |
| 1381 | 24798100 | -7 |
| 1382 | 121285788 | -7 |
| 1383 | 24373321 | -7 |
| 1384 | 4245064 | -7 |
| 1385 | 14737914 | -7 |
| 1386 | 24791476 | -7 |
| 1387 | 56317236 | -7 |
| 1388 | 3715971 | -7 |
| 1389 | 22403156 | -7 |
| 1390 | 85267867 | -7 |
| 1391 | 7973699 | -7 |
| 1392 | 24785860 | -7 |
| 1393 | 85145978 | -7 |
| 1394 | 56318216 | -7 |
| 1395 | 4258242 | -7 |
| 1396 | 144098119 | -7 |
| 1397 | 85269900 | -7 |
| 1398 | 14725684 | -7 |
| 1399 | 17433313 | -7 |
| 1400 | 26652588 | -7 |
| 1401 | 56318091 | -7 |
| 1402 | 26529073 | -7 |
| 1403 | 29216672 | -7 |
| 1404 | 87334730 | -7 |
| 1405 | 862633 | -7 |
| 1406 | 7999806 | -7 |
| 1407 | 85200036 | -7 |
| 1408 | 49828819 | -7 |
| 1409 | 14734328 | -7 |
| 1410 | 11534871 | -7 |
| 1411 | 124894792 | -7 |
| 1412 | 14744389 | -7 |
| 1413 | 49736869 | -7 |
| 1414 | 57259296 | -7 |
| 1415 | 49679890 | -7 |
| 1416 | 49678864 | -7 |
| 1417 | 24806065 | -7 |
| 1418 | 49821613 | -7 |
| 1419 | 24800385 | -7 |
| 1420 | 49736789 | -7 |
| 1421 | 49733718 | -7 |
| 1422 | 17441979 | -7 |
| 1423 | 24804932 | -7 |
| 1424 | 24412891 | -7 |
| 1425 | 57258518 | -7 |
| 1426 | 3716549 | -7 |
| 1427 | 49728819 | -7 |
| 1428 | 24829229 | -7 |
| 1429 | 99454261 | -7 |
| 1430 | 14723770 | -7 |
| 1431 | 144097857 | -7 |
| 1432 | 49815745 | -7 |
| 1433 | 26537603 | -7 |
| 1434 | 3717706 | -7 |
| 1435 | 24827010 | -7 |
| 1436 | 26626004 | -7 |
| 1437 | 17453583 | -7 |
| 1438 | 24832015 | -7 |
| 1439 | 858976 | -7 |
| 1440 | 24825031 | -7 |
| 1441 | 26537770 | -7 |
| 1442 | 17468276 | -7 |
| 1443 | 3716648 | -7 |
| 1444 | 26646409 | -7 |
| 1445 | 49730143 | -7 |
| 1446 | 3712495 | -7 |
| 1447 | 26640842 | -7 |
| 1448 | 24707817 | -7 |
| 1449 | 24326543 | -7 |
| 1450 | 57266657 | -7 |
| 1451 | 26539920 | -7 |
| 1452 | 26541598 | -7 |
| 1453 | 26729774 | -7 |
| 1454 | 24784991 | -7 |
| 1455 | 49729972 | -7 |
| 1456 | 24799276 | -7 |
| 1457 | 24837508 | -7 |
| 1458 | 26648199 | -7 |
| 1459 | 26666005 | -7 |
| 1460 | 24820603 | -7 |
| 1461 | 24373635 | -7 |
| 1462 | 49820388 | -7 |
| 1463 | 144097881 | -7 |
| 1464 | 24789088 | -7 |
| 1465 | 4241351 | -7 |
| 1466 | 860317 | -7 |
| 1467 | 4247254 | -7 |
| 1468 | 24786336 | -7 |
| 1469 | 49675621 | -7 |
| 1470 | 124948262 | -7 |
| 1471 | 24785462 | -7 |
| 1472 | 22407283 | -7 |
| 1473 | 26614518 | -7 |
| 1474 | 24409746 | -7 |
| 1475 | 860594 | -7 |
| 1476 | 26633305 | -7 |
| 1477 | 24389080 | -7 |
| 1478 | 24374501 | -7 |
| 1479 | 26630764 | -7 |
| 1480 | 864433 | -7 |
| 1481 | 17437583 | -7 |
| 1482 | 17445855 | -7 |
| 1483 | 26660224 | -7 |
| 1484 | 26626148 | -7 |
| 1485 | 17462306 | -7 |
| 1486 | 24785821 | -7 |
| 1487 | 24830040 | -7 |
| 1488 | 56322842 | -7 |
| 1489 | 49713860 | -7 |
| 1490 | 99456675 | -7 |
| 1491 | 17471175 | -7 |
| 1492 | 4250791 | -7 |
| 1493 | 103060939 | -7 |
| 1494 | 24327512 | -7 |
| 1495 | 99360936 | -7 |
| 1496 | 17449424 | -7 |
| 1497 | 17405698 | -7 |
| 1498 | 24798746 | -7 |
| 1499 | 49642231 | -7 |
| 1500 | 56317127 | -7 |

**Table S1.** List of compounds obtained from virtual screening against SmHDAC8 within the binding energy range of -9.5 to −7 kcal/mol.

**
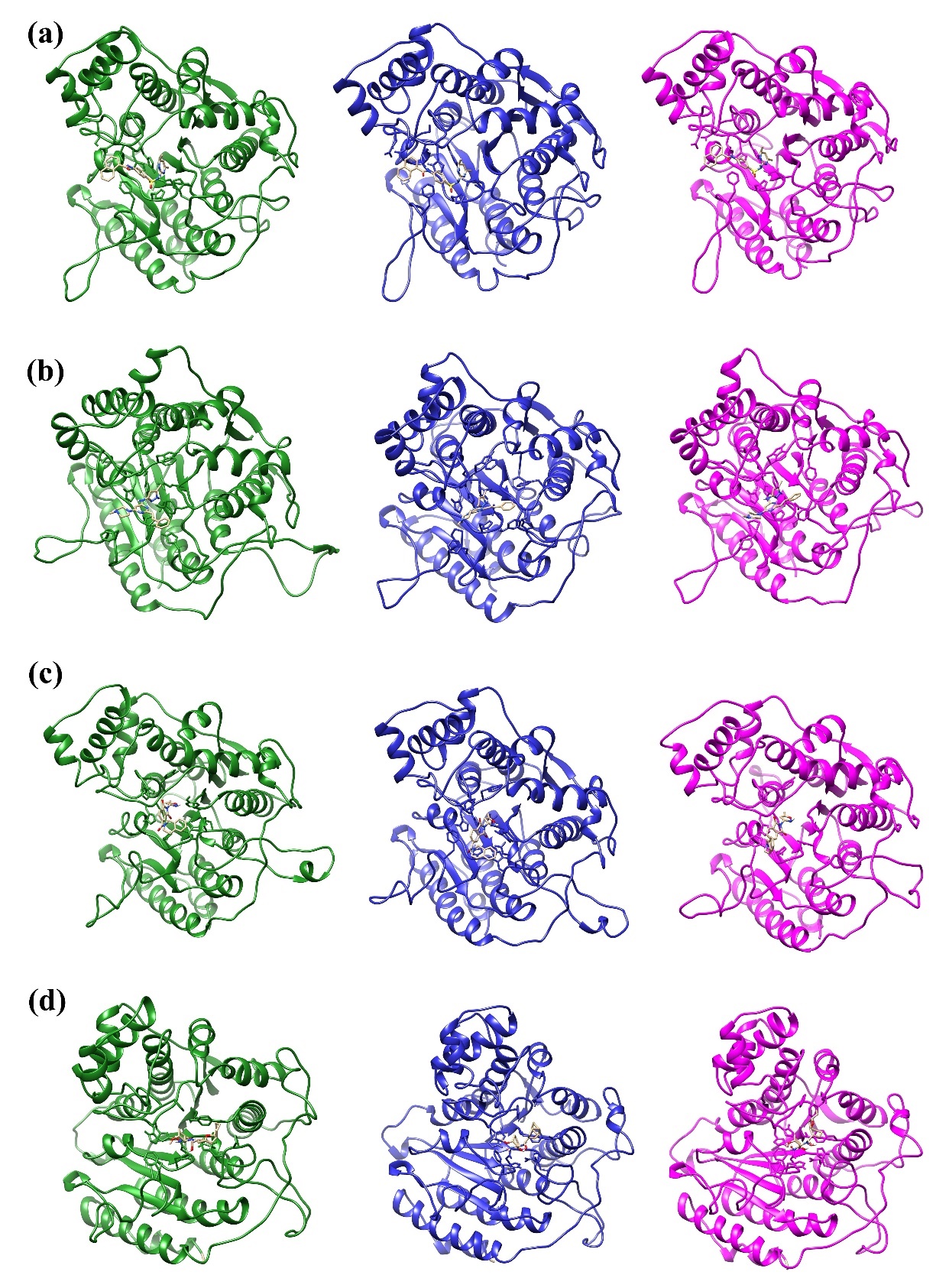
**

**Figure S1 –** Representative low-energy conformations of ligand-bound protein complexes extracted from the free energy landscape (FEL). (a) 24280440, (b) 24374890, (c) 137276022, and (d) the control


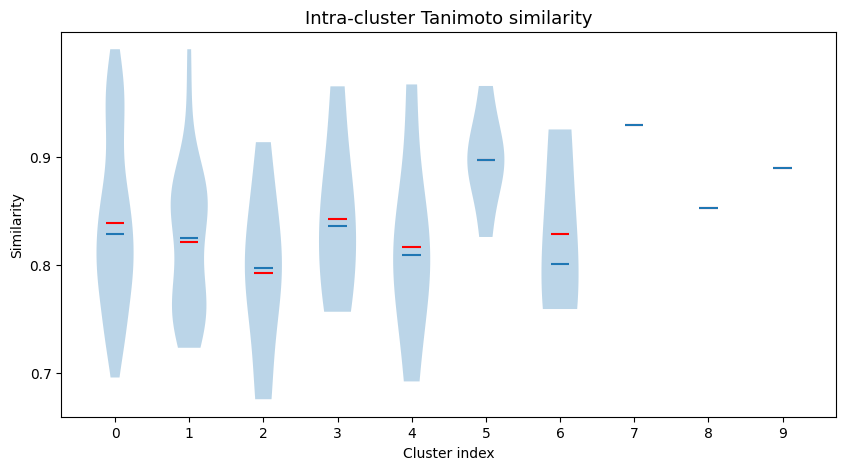


**Figure S2**. Violin plot showing intra-cluster Tanimoto similarity distributions for each cluster (indexed 0–9). The width of each violin represents the density of similarity values within the cluster. Horizontal bars indicate the mean (red) and median (blue) similarities. Higher similarity scores across clusters suggest that structurally similar molecules were effectively grouped during clustering


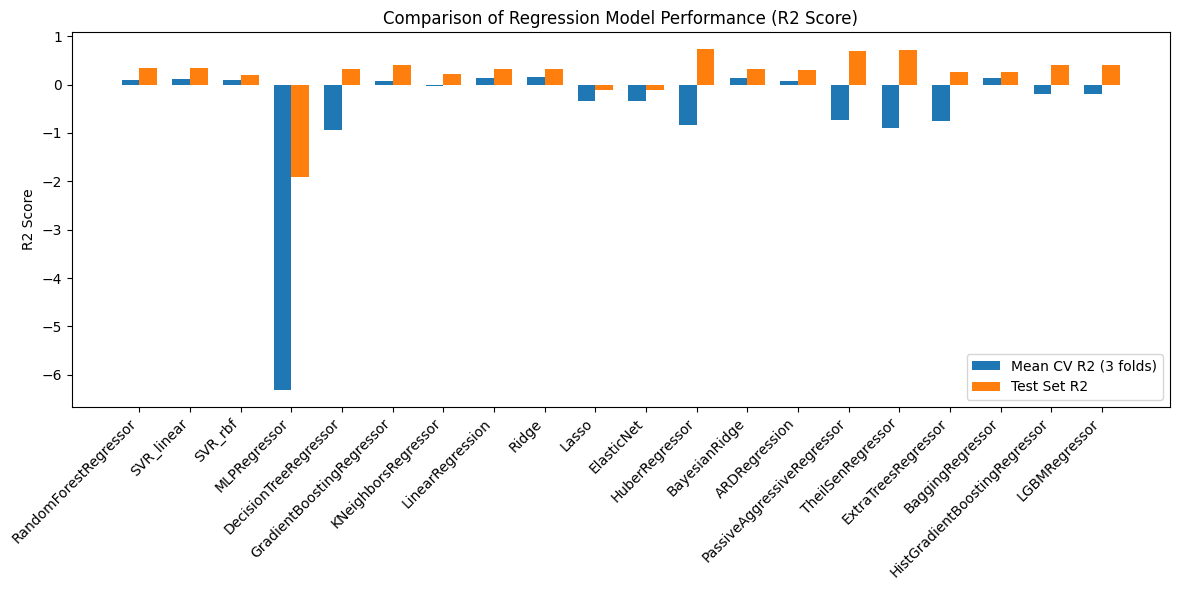


**Figure S3**. Comparison of R² scores across 22 regression models using cross-validation and independent test sets.


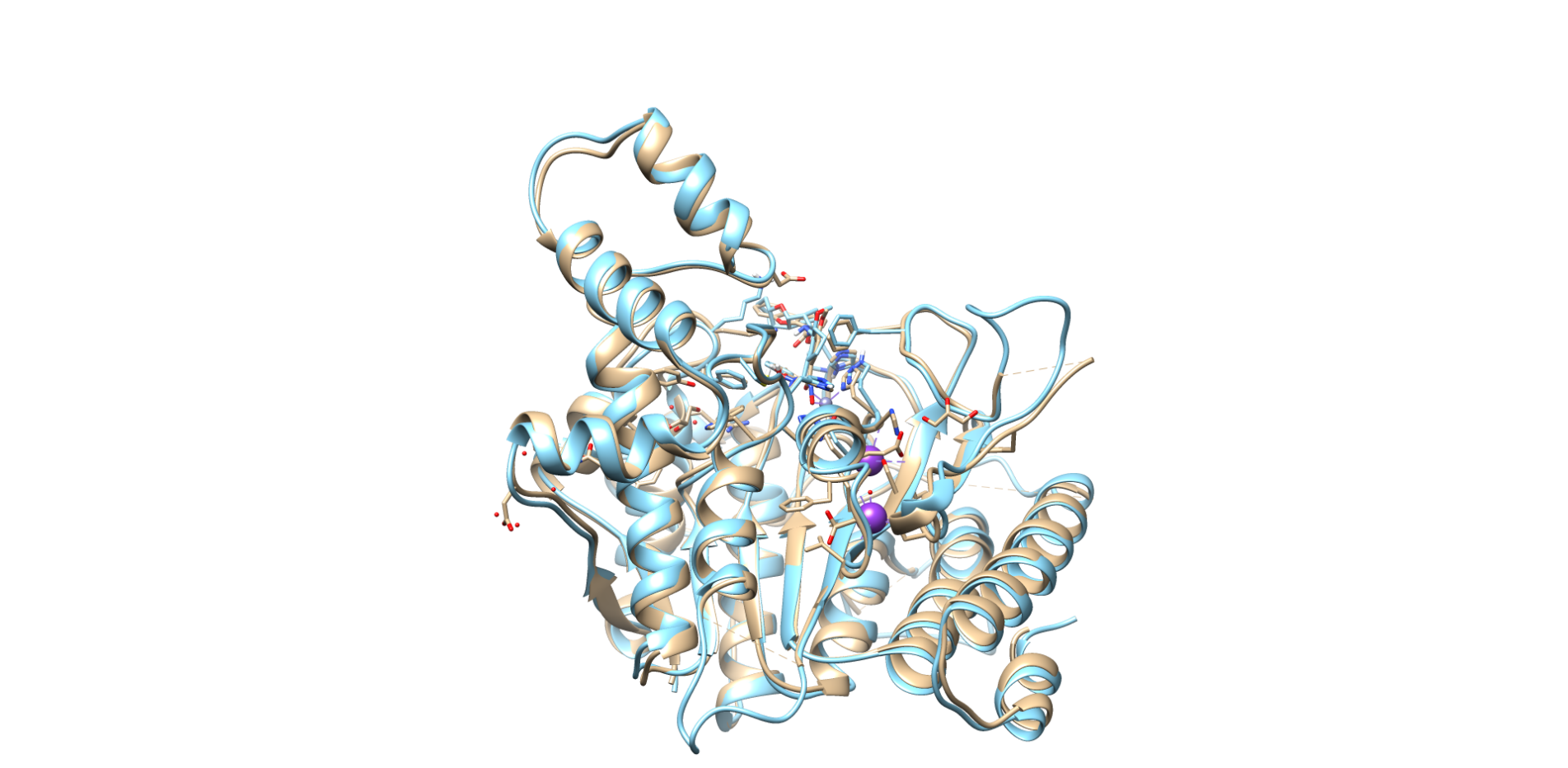



**Figure S4:** Superimposition of the experimental (brown) and redocked (cyan) poses of the co-crystallized SmHDAC8 inhibitor (PDB ID: 7P3S), showing RMSD = 0.7 Å.

**Figure S5:** RMSD of the apo-SmHDAC8 backbone over 500 ns.

**

Figure S6:** RMSF of the apo-SmHDAC8 backbone over 500 ns**.**
